# Training bias and sequence alignments shape protein–peptide docking by AlphaFold and related methods

**DOI:** 10.1002/pro.70331

**Published:** 2025-10-13

**Authors:** Lindsey Guan, Amy E. Keating

**Affiliations:** ^1^ Graduate Program in Computational and Systems Biology Massachusetts Institute of Technology Cambridge Massachusetts USA; ^2^ Department of Biology Massachusetts Institute of Technology Cambridge Massachusetts USA; ^3^ Department of Biological Engineering Massachusetts Institute of Technology Cambridge Massachusetts USA; ^4^ Koch Institute for Integrative Cancer Research Massachusetts Institute of Technology Cambridge Massachusetts USA

**Keywords:** benchmarking, interpretability, machine learning, protein‐peptide interactions, structure prediction

## Abstract

Protein‐peptide interactions mediate many biological processes. Accurate structural models of protein‐peptide complexes, determined by experiment or computational prediction, are essential for understanding function and designing interaction inhibitors. AlphaFold2‐Multimer (AF2‐Multimer), AlphaFold3 (AF3), and related models such as Boltz‐1 and Chai‐1 can predict protein‐peptide binding geometry, often with high accuracy. Using a dataset of experimentally resolved structures, we analyzed the performance of these four structure prediction models to understand how they work. We found evidence of bias for previously seen structures, indicating that models struggle to generalize to novel proteins or binding sites. We tested how models use the protein and peptide multiple sequence alignments (MSAs), which are often shallow or of poor quality for peptide sequences. We found weak evidence that models use coevolutionary information from paired MSAs, but both the protein and peptide unpaired MSAs contribute to prediction accuracy. Our work highlights the promise of deep learning for peptide docking and the importance of diverse representation of interface geometries in the training data for optimal prediction performance.

## 1 INTRODUCTION

Many protein–protein interactions involve a well‐folded domain engaging a smaller peptide (Petsalaki & Russell, 2008). However, the Protein Data Bank (PDB) captures only a small fraction of these interactions, which are often low‐affinity and dynamic. Protein structure‐prediction methods, such as AlphaFold2 (AF2), AF2‐Multimer, and AlphaFold3 (AF3) (Abramson et al., 2024; Evans et al., 2022; Jumper et al., 2021), can accurately predict many protein–protein complex structures, and benchmarks for protein‐peptide docking have shown that performance is also promising for these complexes. AF2, which was trained on only single‐chain structures, predicted 51% of domain‐peptide complexes with <2.5 Å interface backbone RMSD when “hacked” to predict the structure of complexes by fusing a protein to its peptide partner using a poly‐glycine linker (Tsaban et al., 2022). Ko et al. found that 50% of protein‐peptide complexes, fused with a glycine linker, were predicted with <2 Å peptide backbone RMSD (Ko & Lee, 2021). AF2‐Multimer, which was trained on single chains and multi‐chain complexes, encodes chains separately and predicts structures for complexes without requiring a linker. Johansson‐Åkhe et al. found that AF2‐Multimer outperformed other established methods and achieved an acceptable prediction in 60% of test cases (Johansson‐Åkhe & Wallner, 2022). A summary of methods and success rates reported in previous studies can be found in Table S1.

Because peptides are shorter than globular proteins and evolve under functional constraints that can be difficult to capture in multiple‐sequence alignments (MSAs) (Chica et al., 2008; Davey et al., 2012), they represent a special case for models that heavily rely on an MSA. Since the release of AF2, structure prediction models have been developed that do not use an MSA, such as ESMFold and OmegaFold (Lin et al., 2023; Wu et al., 2022). However, MSA‐based models remain the most performant for predicting protein complex structures (Zhu et al., 2023). Bryant et al. observed that AF2‐Multimer predicted bacterial protein–protein complexes more accurately than complexes from eukaryotes, which they attributed to having more sequenced bacterial genomes in which to identify homologs (Bryant et al., 2022; Green et al., 2021). They observed that the fraction of correctly predicted structures increased with a larger number of non‐redundant sequences in the MSA. For predictions involving multiple chains, cross‐chain coevolutionary information is available to structure prediction models through sequence pairing during MSA construction. Sequences from the same species are paired in each row of the MSA, followed by the unpaired sequences on the block diagonal. Whether and how AF2‐Multimer and newer models use the MSA input, including MSA pairing, to make accurate predictions for protein‐peptide complexes remains unclear.

Structure prediction models form the basis of many methods developed for the discovery or design of protein‐binding peptides (Bennett et al., 2023; Bret et al., 2024; Goudy et al., 2023; Lee et al., 2024). To inform further development of such methods, we tested the requirements for accurate deep learning‐based protein‐peptide complex predictions. We benchmarked AF2 (trained on single‐chain proteins), AF2‐Multimer, AF3, Boltz‐1, and Chai‐1 on a set of protein‐peptide complexes, reproducing and extending earlier findings of good performance (Abramson et al., 2024; Chai Discovery team et al., 2024; Evans et al., 2022; Wohlwend et al., 2024). We then sought to explain the observed range of prediction accuracies through an assessment of sequence and structural similarity to the training set and found a strong dependence of prediction accuracy on overlap with the training set. We investigated the ability of models to process MSA statistics and found that inter‐chain coevolution is weakly related to prediction success for protein‐peptide and protein–protein complexes. However, information in the unpaired MSAs for both protein and peptide chains contributes to prediction accuracy, and we found evidence that AF2‐Multimer can derive inter‐chain information from unpaired MSAs. The protein MSA contains information that guides binding‐site prediction, and the peptide MSA improves both binding‐site and peptide conformation prediction. Strikingly, good predictions can be made for many complexes when the peptide sequence is masked.

## 2 RESULTS

### 2.1 State‐of‐the‐art methods accurately predict most protein‐peptide complex structures

**FIGURE 1:**
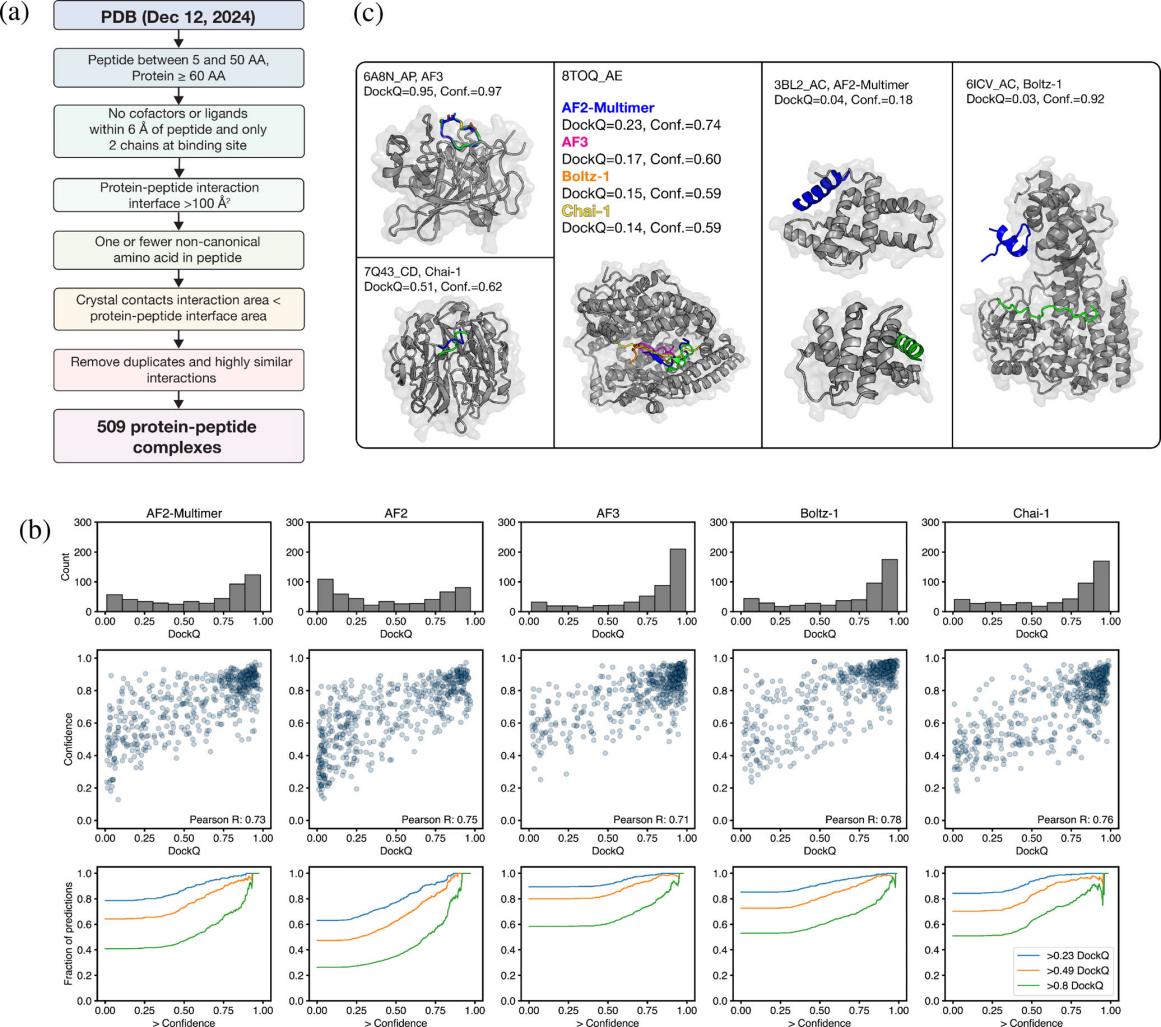
Structure prediction methods accurately dock most test‐set peptides. (a) Curating protein‐peptide complexes. Applying filters and clustering resulted in a non‐redundant test set of 509 protein‐peptide complexes. (b) Top: Prediction accuracy (DockQ) and confidence (ipTM + pTM) of all models on the test‐set structures; bottom: Cumulative distributions showing DockQ as a function of ipTM + pTM. The vertical axis tracks the fraction of samples with DockQ greater than the value indicated in the legend, as a function of the confidence cutoff indicated on the horizontal axis. (c) Example predictions. For 6A8N_AP, 7Q43_CD, 3BL2_AC, and 6ICV_AC, predictions are in blue and native peptides are in green. For 8TOQ_AE, predictions are in blue, magenta, orange, and yellow, and the native peptide is in green. For all except 3BL2_AC, only the experimental structure of the protein is shown.

To curate protein‐peptide complexes for evaluation, we filtered structures from the PDB by peptide length, presence of cofactors, and crystal contacts near the peptide binding site (Figure 1a) (Martins et al., 2021). We clustered complexes according to the similarity of the protein structure and then by the similarity of the bound peptide conformation. We aimed to balance the removal of redundant entries with maintaining sufficient examples for statistical power and to capture variability in model performance, even across structurally similar complexes (Methods, "Dataset"). We made predictions using unpaired MSAs and no templates, took the top‐ranked structure out of five predictions, and calculated DockQ values between predicted and experimental structures. DockQ is a linear combination of the peptide backbone RMSD, interface backbone RMSD, and number of recovered native contacts normalized to the range 0–1, with 1 representing perfect recovery (Basu & Wallner, 2016; Mirabello & Wallner, 2024). We use DockQ performance categories described by Basu et al., which map closely to the “acceptable,” “medium,” and “high‐quality” predictions used in the CAPRI assessment (Janin, 2005). For all methods, many predictions for our test structures were of high quality (DockQ >0.80), with the peptide binding pose mostly correct (Figure 1b and S1a). This performance is consistent with previous studies benchmarking AF2‐Multimer for protein‐peptide structure prediction (Table S1) (Ko & Lee, 2021; Tsaban et al., 2022). Some predictions were medium (DockQ >0.49) or poor (DockQ <0.23); in such cases, the peptide orientation or the binding site was incorrect.

Five complexes predicted with varying accuracy are shown in Figure 1c. PDB 6A8N chains A and P (henceforth 6A8N_AP), the structure of a urokinase‐type plasminogen activator in complex with a small cyclic peptide, provides an example of a highly accurate prediction with AF3: The modeling captured the correct binding interaction and the disulfide bond cyclizing the peptide (PDB, 2019a; Wang et al., 2019). 7Q43_CD, the structure of HERC2 RLD2 bound to a DOCK10 DXDKDED motif, is an example of a medium accuracy prediction with Chai‐1, where the binding site is correct but the peptide orientation is not (PDB, 2022). For 8TOQ_AE, the structure of an ACE2‐peptide complex, all models predicted the peptide orientation incorrectly (Bedding et al., 2024; PDB, 2024a). We found a few instances of models failing to accurately predict the protein structure, such as the AF2‐Multimer prediction of 3BL2_AC, a structure of Bcl‐2 family protein M11 bound to a Beclin1 peptide (Ku et al., 2008; PDB, 2008). Finally, many instances of poor predictions were the result of an incorrect binding‐site prediction, such as the high‐confidence yet incorrect Boltz‐1 prediction of 6ICV_AC, the structure of SETD3 bound to actin (Guo et al., 2019; PDB, 2019b). In addition to DockQ, which is based on a backbone‐only RMSD calculation, we also defined criteria to reflect what we refer to as atomically accurate predictions: >90% of native contacts recovered, no clashes, peptide all‐atom RMSD <2 Å, and interface all‐atom RMSD <3 Å. 11%–34% of predictions were accurate to this degree, with AF3 achieving the best performance (Figure S1b).

The AF2‐Multimer confidence score is reported to be an excellent scoring function for multimeric structure predictions. Models that used the highest‐ranking prediction after extensive structural sampling surpassed other methods at CASP15 (Wallner, 2023). For our protein‐peptide test set, the Pearson correlation coefficient between confidence (ipTM+pTM) and DockQ was >0.7 for all methods (Figure 1b, middle). Of predicted complexes with ipTM+pTM >0.75, 66%–77% were high‐quality and 89%–95% were medium‐ or high‐quality across all models (Figure 1b, bottom). Low‐confidence structures were rarely accurate (i.e., there was a low false negative rate), but all models generated high‐confidence yet incorrect predictions (i.e., there was a higher false positive rate). Similar to ipTM+pTM, the correlation between interaction predicted aligned error (iPAE) and DockQ ranged from −0.62 to −0.75 for all methods (Figure S2a). We also calculated pDockQ (Bryant et al., 2022), which yielded a worse correlation with DockQ than ipTM+pTM or iPAE (Figure S2b). We found a weak correlation between chain length and accuracy for all models (Figure S3).

To assess how well models predict the secondary structure adopted by the bound peptide, irrespective of docking accuracy, we used DSSP to define secondary structure in the experimental and predicted peptide structures (Joosten et al., 2011; Kabsch & Sander, 1983). All models predicted more residues to be α‐helical and less loop‐like than the experimental structures (Figure S4a). Complexes where the interface is composed mostly of loop‐like residues were, on average, predicted less successfully than complexes with regular secondary structure (Figure S4b).

### 2.2 Prediction accuracy is lower for previously unseen complexes

**FIGURE 2:**
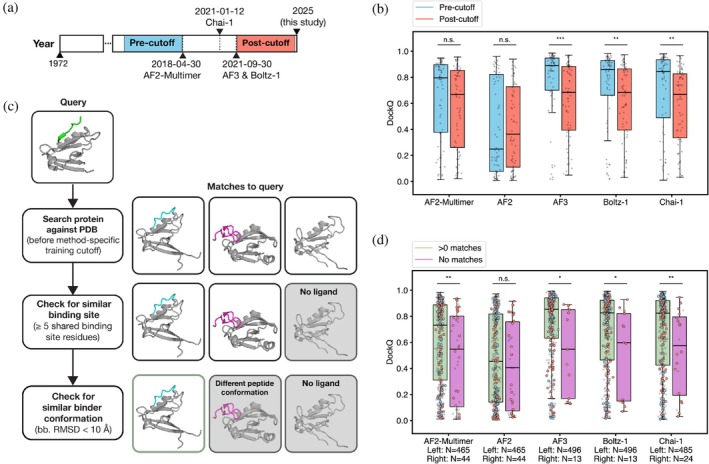
Prediction accuracy improves with similarity to training set examples. (a) Sets of pre‐cutoff and post‐cutoff structures were selected based on the earliest pre‐training cutoff (AF2‐Multimer) and the latest post‐training cutoff (AF3/Boltz‐1). 68 post‐training structures and the latest 68 pre‐training structures were chosen. (b) Distribution of DockQ scores for predictions where the PDB entry was available for training (pre‐cutoff) and where it was unseen (post‐cutoff), *N* = 68 for each. (c) Procedure for identifying binding‐site matches in the PDB for a target complex (“Query”) based on method‐specific training data cutoffs (Methods, “Search for interaction matches in the PDB”). Structures on the right are hits, with grayed‐out structures filtered out at each step in the pipeline. (d) Distribution of DockQ for predictions where the binding site had at least one match in the training set versus not represented in the training set. Data points are colored corresponding to their presence in the pre‐cutoff or post‐cutoff sets, consistent with Figure 2b. Gray points are test set structures not included in Figure 2b. (Two‐sided Mann–Whitney *U*‐test, *p < 0.05, **p < 0.01, ***p < 0.001).

We tested how prediction accuracy relates to the similarity of the target complex to the training set. Previous work reported similar AF2 performance for protein‐peptide structures released before and after the AF2 training cutoff (Bret et al., 2024; Tsaban et al., 2022). To examine whether there is a dependency for AF3, Boltz‐1, and Chai‐1, we constructed a post‐training cutoff set and a pre‐training cutoff set, using the cutoff date for the most recently trained models, AF3 and Boltz‐1. The post‐cutoff set consists of 68 structures, and the pre‐cutoff set consists of the latest 68 structures released before the training cutoff date (Methods, “Pre‐training and post‐training cutoff split”) (Figure 2a). AF3, Boltz‐1, and Chai‐1 performed worse on the post‐cutoff structures than the pre‐cutoff structures (*p* = 4.4E−3 to 1.2E−4; Figure 2b), but there was no significant difference in the performance of AF2 or AF2‐Multimer on these sets of structures (*p* = 0.10 to 0.91). When evaluating only post‐cutoff structures, all models, except AF2, had comparable performance, with median DockQ ranging from 0.67 to 0.69. Strikingly, for AF3, Boltz‐1, and Chai‐1, only 6%–13% of post‐cutoff predictions were atomically accurate, while 22%–38% of pre‐cutoff predictions were atomically accurate (Figure S5b), indicating that high accuracy may be supported by memorization of structural details in the training set.

Defining a test set using a cutoff date does not eliminate strong similarities between training and test structures. In a more stringent assessment of training and test‐set overlap, for each test complex, we searched for homologous proteins (by sequence or structure) in the training set and checked whether matching structures were bound to a partner structurally similar to the peptide (<10 Å RMSD) (Figure 2c, Methods, “Search for interaction matches in the PDB”). In cases where this was true, we designated the training set complex as a binding‐site match to the target complex. For all models except AF2, binding sites with at least one binding‐site match in the training set were predicted more accurately than interactions that were not represented (two‐sided Mann–Whitney *U*‐test, *p* < 0.05, Figure 2d). AF2 did not exhibit the same level of training‐set bias as the other models, which was expected because AF2 was not trained on protein complexes. Nevertheless, AF2 prediction performance was worse than that of the other methods, and we did not include this method in subsequent analyses.

We saw little correlation between the number of binding‐site matches in the training set and DockQ, although highly represented complexes were almost always predicted well. Across models, 89%–100% of complexes with more than 200 binding‐site matches had DockQ ≥ 0.49 (Figure S5a). Among complexes with no binding‐site matches in the training set, none of the structures predicted by AF3, Boltz‐1, or Chai‐1 were atomically accurate (Figure S5b). Training‐set bias does not arise from memorization of the bound peptide structure; predictions for the peptide chain, alone, were dissimilar from the bound peptide structure, with median TM‐scores of 0.27–0.37 (Figure S5c).

**FIGURE 3:**
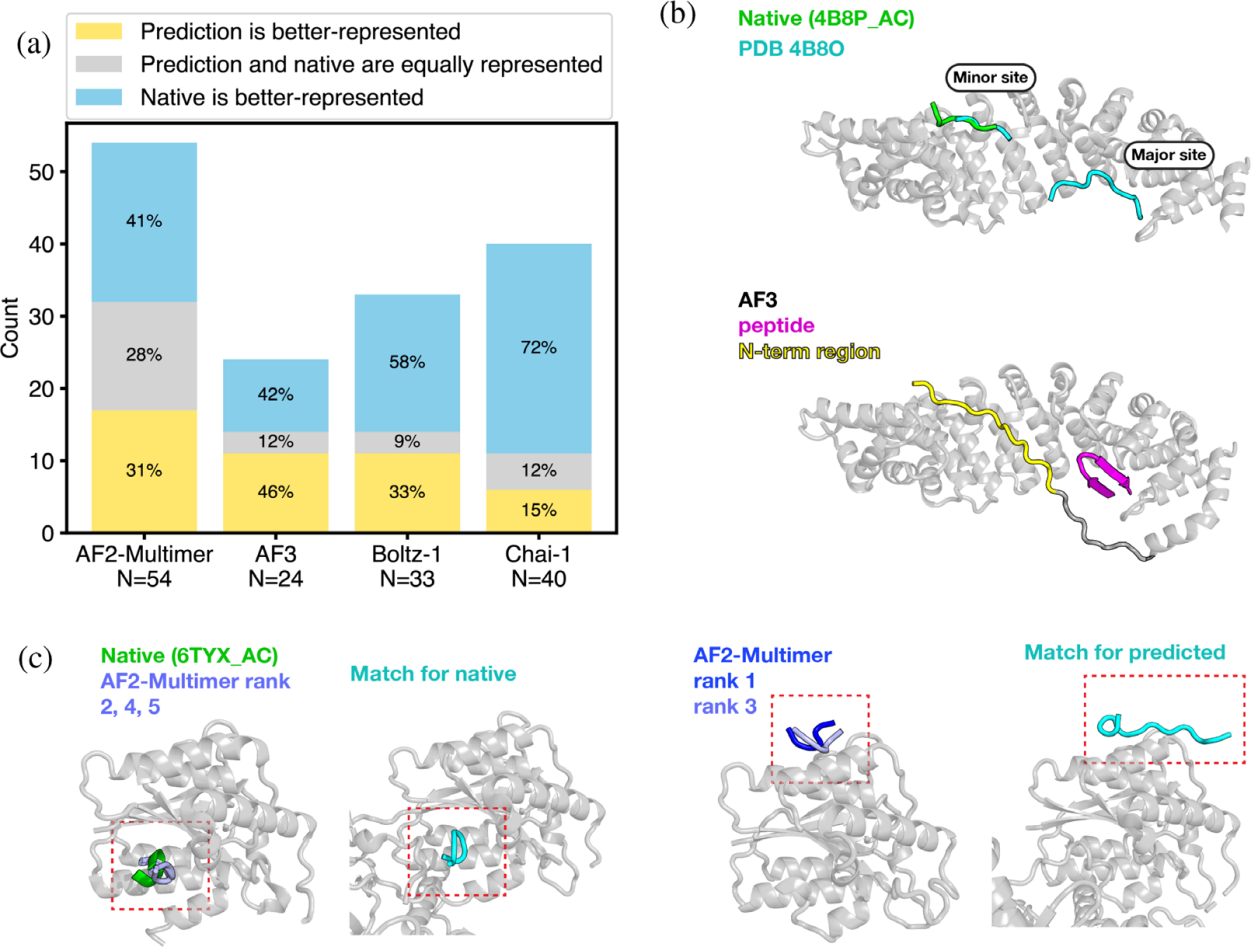
Some incorrect predictions correspond to structures seen frequently in the training data. (a) Binding site representation for the native and predicted binding sites for docking predictions with protein TM‐score >0.6 and DockQ <0.23 (poor), that is, cases for which docking failed despite an acceptable structure prediction for the protein. (b) Top: Experimental structure for 4B8P_AC in green, with PDB 4B8O shown in cyan to illustrate the major and minor NLS‐binding regions of importin α; Bottom: AF3 prediction for 4B8P_AC. (c) Left: Native structure for 6TYX_AC with the second‐, fourth‐, and fifth‐ranked AF2‐Multimer predictions (conf. = 0.47, 0.44, 0.43, respectively); middle left: A representative binding site match for the native complex, PDB 6ERG; middle right: The top‐ and third‐ranked AF2‐Multimer predictions (conf. = 0.48, 0.46, respectively); right: A representative binding site match for the predicted complex, PDB 6ERF (Nemoz, Legrand, et al., 2018a; Nemoz, Legrand, et al., 2018b; Nemoz, Ropars, et al., 2018).

We explored the possibility that incorrect predictions arise because they resemble binding modes seen frequently in the training data. We analyzed complexes for which the protein structure was predicted with TM‐score >0.6 but peptide docking was poor (DockQ <0.23). For a substantial fraction of cases (e.g., 42% for AF3), the predicted interface had more binding‐site matches in the training set than the experimentally observed interface did (Figure 3a). The ARM domain of importin α presents an interesting case. This domain has two nuclear‐localization‐signal (NLS) binding sites, a major and minor site (Figure 3b) (Kosugi et al., 2009). Both binding sites on 4B8P_A are well‐represented in the training data, with 107 binding‐site matches for the major site and 73 matches for the minor site. In 4B8P_AC, the NLS peptide binds specifically to the minor site of *Oryza sativa* importin α, though AF3 predicted the peptide to bind in the major site and, interestingly, docked the N‐terminus of the ARM domain in the minor site (Figure 3b) (Chang et al., 2012; PDB, 2013). For 6TYX_AC, the structure of the Ku80 vWA domain bound to a Ku‐binding motif, the experimental and predicted binding sites had 4 and 6 matches, respectively (Kim et al., 2020; PDB, 2019b). Among the five predictions from AF2‐Multimer, the top‐ranked and third‐ranked predictions placed the peptide in the same incorrect binding site, while the second‐, fourth‐, and fifth‐ranked structures placed the peptide in the experimentally observed site (Figure 3c).

### 2.3 Models do not rely on MSA sequence pairing for protein‐peptide predictions

Despite the strong association between high prediction accuracy and target complex–training set overlap, there were nevertheless correct predictions for complexes that lacked any binding‐site matches in the training dataset (Figure 2d). There were also incorrect predictions for complexes with structural similarity to the training data. This motivated the question of what information powers predictions across the entire test set.

Previous studies have shown that MSA‐based models rely on the MSA to make accurate predictions (Jumper et al., 2021). In our tests, too, models rarely made accurate protein‐peptide structure predictions when we ablated both the protein and peptide MSAs (Figure S6a). Most structures for which accurate predictions were made without an MSA were represented in the training set. The few novel complexes that were predicted correctly had interfaces with canonical secondary structure elements (e.g., the peptide forms a strand in a β‐sheet). One was 8FG6_BA, which is a *de novo* designed complex of an amyloidogenic peptide and an amyloidogenic peptide trap (Sahtoe et al., 2024). This successful prediction is consistent with previous reports that *de novo* designed proteins can be predicted without an MSA (Figure S6b) (Anishchenko et al., 2021; Goverde et al., 2023; Pereira et al., 2021).

We explored the importance of the peptide MSA and protein‐peptide MSA pairing by species. Although most complexes had a deep MSA for the protein, most peptide sequences had sparse MSAs. Only 168/509 complexes had a non‐empty peptide MSA, and 114/509 had a peptide MSA with at least 50 sequences (Figure S7a). The results described so far used these default MSAs without pairing by species and thus without access to protein‐peptide coevolutionary information. When we tried to construct paired MSAs using default methods, only two complexes resulted in a paired MSA with at least 50 sequences; for most complexes, the paired MSA was empty (Figure S7a).

**FIGURE 4:**
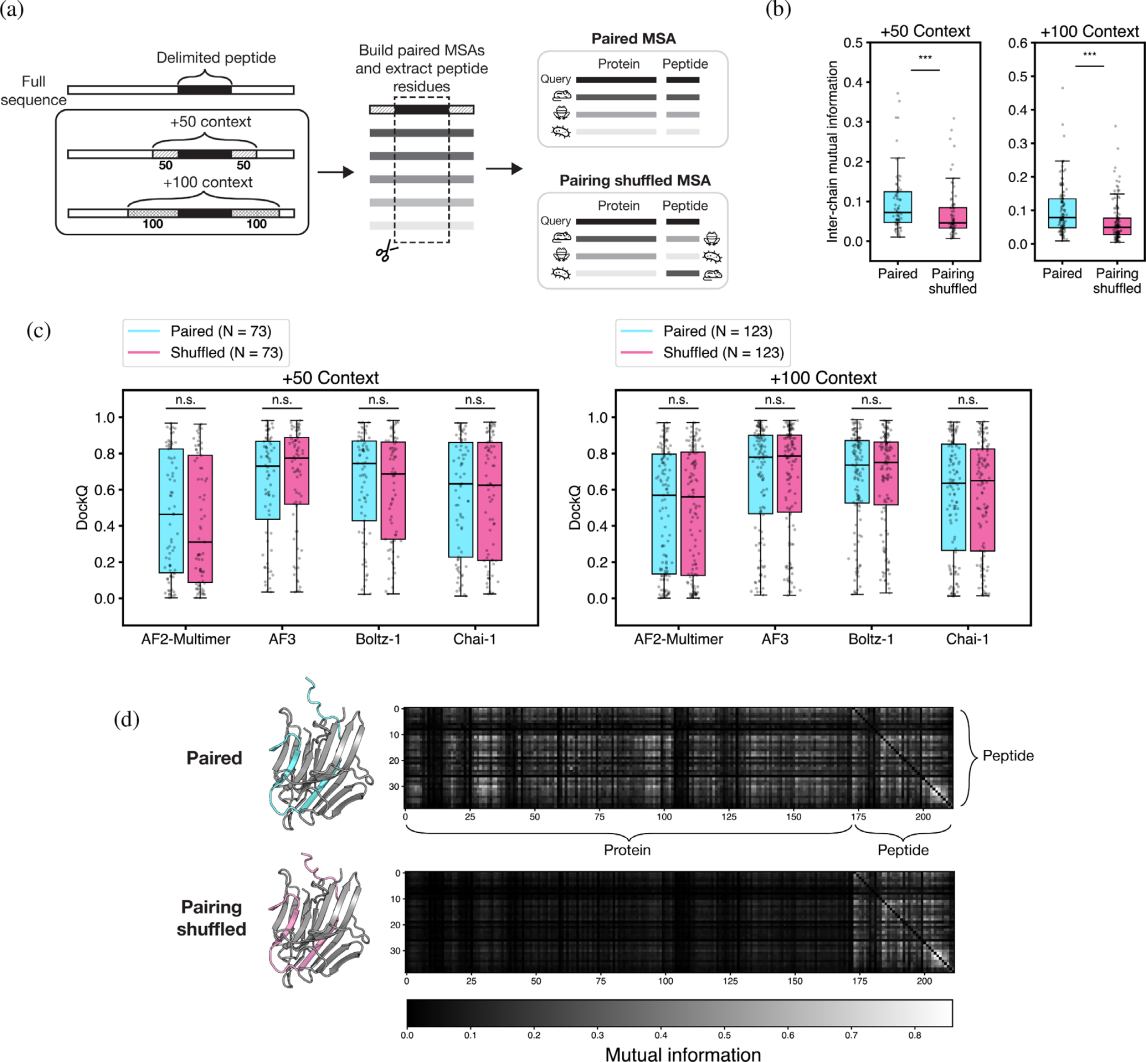
MSA pairing does not improve prediction performance. (a) Procedure for constructing MSAs using additional peptide context. Paired and shuffled MSAs were constructed using the peptide sequence with an additional 50 or 100 residues on either side of the delimited peptide. (b) Distribution of mutual information (MI) and DockQ scores for predictions made with paired (cyan) versus shuffled paired (pink) MSAs (Wilcoxon signed‐rank test, *p < 0.05, **p < 0.01, ***p < 0.001). (c) An example is shown for 3TRS_BA with 100‐residue context, predicted with AF2‐Multimer. The structure prediction is shown for the paired MSA (cyan, DockQ = 0.86) and shuffled MSA (pink, DockQ = 0.82). MI for peptide residues with every other residue in the query (protein + peptide) is shown in the heatmap.

To test whether models can use coevolutionary information, we constructed deeper paired MSAs for cases where the peptide could be mapped to a canonical UniProt entry. We extracted the protein sequence from which the peptide sequence was derived and included 50 or 100 flanking residues (“context”) on either side of the PDB‐delimited peptide (Dana et al., 2019) (Methods, “Mapping peptide sequences to the full‐length protein”). These extended sequences were used as the queries when building paired MSAs. Then, for the peptide chain, all MSA columns were removed except those corresponding to the PDB‐delimited peptide before the paired MSA was used as input to the models (Figure 4a). This procedure is similar to approaches used for other protein fragment binding predictions and yielded deeper paired MSAs for many complexes (Figure S7a) (Bret et al., 2024; Lee et al., 2024; Savinov et al., 2025). Shallow or low‐quality MSAs constructed in this manner, as measured by Jensen‐Shannon divergence, were filtered out (Capra & Singh, 2007), leaving 73 populated paired MSAs with 50 residues of context and 123 populated paired MSAs with 100 residues of context. To reduce variability in structure predictions caused by including different unpaired MSA samples across recycles, we only used the paired MSA.

We compared prediction performance using the paired MSAs to predictions made using MSAs containing the same sequences but with protein‐peptide pairings randomized. We calculated inter‐chain mutual information (MI) for the original and shuffled alignments (Gloor et al., 2005; Martin et al., 2005; Tillier & Lui, 2003). As expected, the shuffling procedure lowered the MI in the paired MSAs (Figure 4b). However, there was little difference in prediction accuracy between models, indicating that the coevolutionary signal between chains is not important for successful docking (Figure 4c). In Figure 4d, for complex 3TRS_BA, the heavy and light chains of a peptidase from the fungus *Aspergillus niger* illustrate these trends. Both the paired and shuffled MSA predictions identified the correct binding site, even when the MI between the peptide and protein MSA columns decreased (Figures 4d and S7) (PDB, 2012; Sasaki et al., 2012). This observation held when we assessed the pre‐training cutoff set and post‐training cutoff sets individually (as defined in Figures 2a and S9), though the power of the test was limited by the small number of post‐training cutoff complexes for which we could build paired MSAs. Predictions made with MSA pairings based on 100 residues of context tended to be more accurate than predictions made with MSA pairings based on 50 residues of context, likely due to the deeper MSAs (Figure S7a).

To compare this result using deeper paired MSAs for protein‐peptide complexes with data for protein–protein complexes, which typically have deeper and higher‐quality MSAs, we repeated the MSA shuffling procedure for a set of protein–protein complexes from Bryant et al. (Bryant et al., 2022). We analyzed 340 complexes with paired MSAs containing at least 50 sequences. Both the paired and unpaired MSAs were included in the prediction to ensure that the individual chains folded well (Figure S10b). MSA shuffling resulted in lower inter‐chain mutual information (Figure S10a) and slightly worse performance for AF2‐Multimer and Boltz‐1, whereas no significant effect was observed with AF3 or Chai‐1 (Figure S10b). Although the decreases in performance for AF2‐Multimer and Boltz‐1 were statistically significant, ablating coevolution was only detrimental for a small subset of complexes (Figure S10c). One example of a complex for which the shuffling degraded performance is 4ZHY_BA, the structure of bacterial signaling complex YfiRB, for which AF2‐Multimer failed to recover the correct docked pose after the paired MSA was shuffled (Figure S10d) (Li et al., 2015; PDB, 2016).

### 2.4 The roles of the unpaired protein and peptide MSAs

**FIGURE 5:**
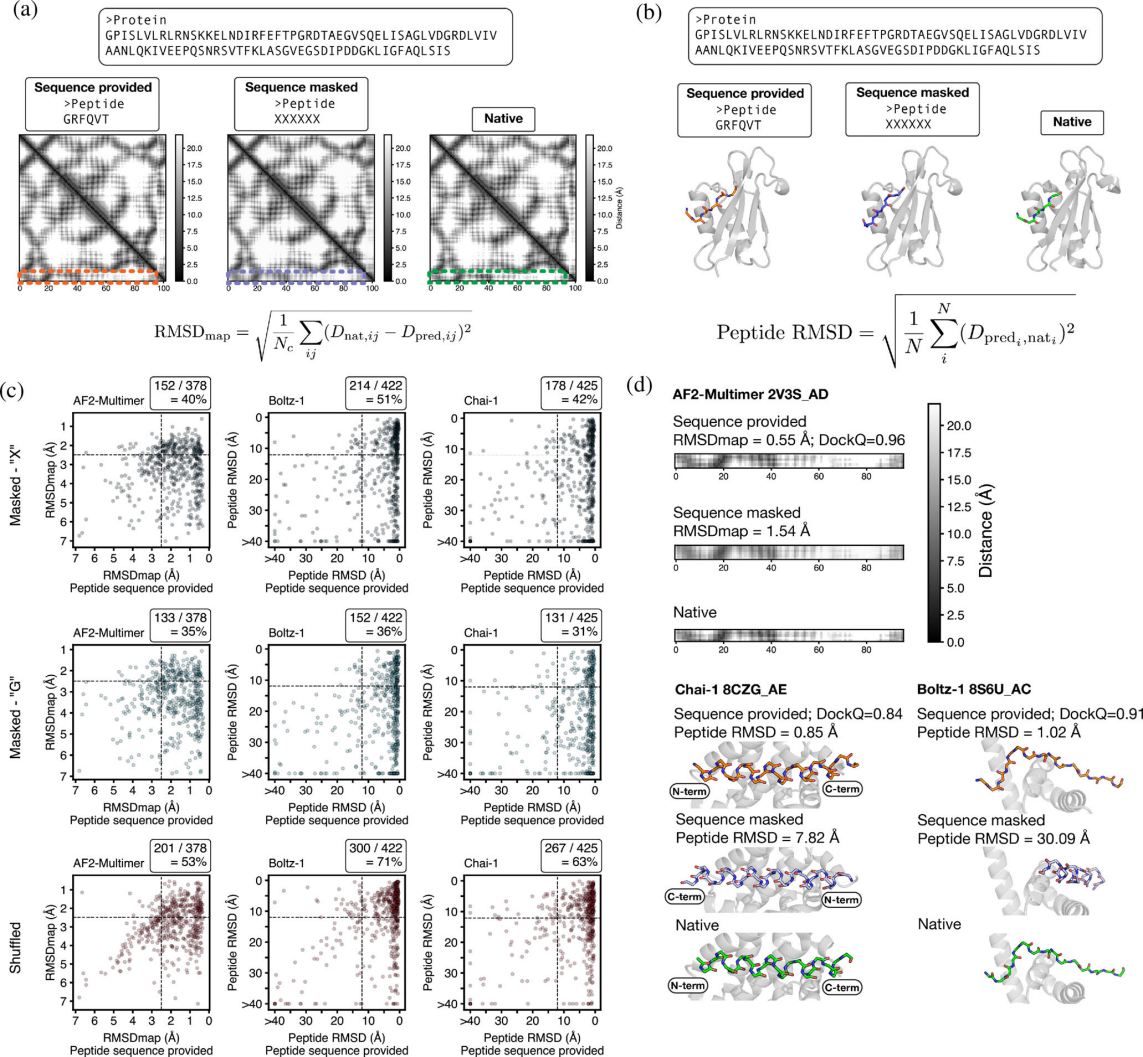
Peptides can be docked without knowledge of the peptide sequences. (a) Procedure for masking the peptide sequence and calculating the distance between native and predicted contact maps (RMSD_map_) for AF2‐Multimer predictions. In the contact maps, interchain contacts are shown in dashed boxes. 2V3S_AD is shown as an example (Villa et al., 2007). $N_{c}$ is the number of pairwise contacts, $D_{\mathrm{nat},\mathrm{ij}}$ is the distance between residues i and j in the native distogram, and $D_{\text{pred},\mathrm{ij}}$ is the distance between residue i and j in the predicted distogram. (b) Procedure for masking the peptide sequence and calculating peptide RMSD for Boltz‐1 and Chai‐1. RMSD is calculated over peptide backbone residues after aligning the protein structures. N is the number of peptide backbone atoms, and $D_{{\text{pred}}_{i},{\mathrm{nat}}_{i}}$ is the distance between atom i in the native and predicted structures. **(c)** RMSD_map_ and peptide RMSD values for predictions made with the peptide sequence provided (x‐axis) and with the peptide sequence modified in three ways (y‐axes): Masked with the token “X,” assigned as poly‐glycine, or randomly shuffled. Fractions reflect the number of successful predictions that remained successful after each sequence modification (i.e., examples in the upper‐right section over all examples to the right of the indicated horizontal axis cutoff) (d) Top: An example of the RMSD_map_ score and the corresponding interchain contact map; Bottom: Two examples of the peptide RMSD score and the corresponding prediction with peptide backbone residues are shown (Aguilar et al., 2023; Devan et al., 2024; PDB, 2023a; PDB, 2024b).

One hypothesis for how deep‐learning models dock peptides onto proteins is that models have learned to identify binding sites on the protein. We tested whether structure‐prediction models have learned to predict binding sites independently of the peptide sequence provided. First, we ablated the peptide sequence using masking with “unknown” tokens, “X” (Figure 5a). We made masked predictions with AF2‐Multimer, Boltz‐1, and Chai‐1. AF3 was not tested because it does not predict structure for masked residues. For Boltz‐1 and Chai‐1, we used the peptide RMSD, calculated after aligning the proteins, to compare predicted peptide backbone structures to the experimental structures (Figure 5b). We considered a peptide RMSD score of <12 Å an acceptable prediction because at this cutoff, 98%–99% of unmasked peptides were predicted with DockQ >0.23 (Figure S11). AF2‐Multimer does not return a structure for a peptide composed of unknown tokens, but it generates a distogram that contains predicted inter‐residue distances. Accordingly, we assessed AF2‐Multimer masked prediction accuracy by comparing contact maps predicted by the distogram head using a new metric, “RMSD_map_” (Figure 5a). RMSD_map_ is based on a comparison of pairwise distances in the predicted and experimental distograms. We considered RMSD_map_ <2.5 Å a docking success: at this cutoff, 93% of unmasked peptides were predicted with DockQ >0.23 (Figure S11).

Of the complexes for which the sequence‐provided prediction was a success, 40%–51% were successfully predicted without use of the peptide sequence (Figure 5c). Examples are shown in Figure 5d to illustrate contact maps and backbone predictions at different RMSD_map_ and peptide RMSD values. We also tried scrambling the sequence of the peptide, maintaining a fixed composition but randomizing the residue order. The number of successes was even greater in this case. Performance degraded, however, when we used glycines instead of mask tokens. We did not find a relationship between presence in the training set and the ability of models to make a successful masked peptide prediction (Figure S12).

To investigate how the protein MSA guides docking, we examined whether sequence conservation explains how models choose binding sites. We annotated the conservation of positions in the protein MSA using Jensen‐Shannon divergence and observed that, as expected, binding‐site residues tended to be more conserved than non‐binding‐site residues (Figure S14a, Methods, “Calculating conservation and hydrophobicity”) (Capra & Singh, 2007). We found that predicted binding‐site residues and binding‐site residues in the experimental structures had very similar average conservation scores (Figure S14b). Specifically, the average conservation of predicted binding sites was not higher than that for the experimental sites, except for AF2‐Multimer predictions, for which the difference was minimal. Figure S14c shows an example where the predicted site is more highly conserved than the native binding site (AF2‐Multimer for 3ICI_AC) and a counterexample where the predicted site is much less conserved (Boltz‐1 for 6ICV_AC). We also tested whether the models selected binding sites by identifying hydrophobic patches on protein structures, particularly in cases where the prediction was poor. However, incorrectly predicted binding sites did not have higher average hydrophobicity than native binding sites (Figure S15).

For AF2, the MSA and the Evoformer have been hypothesized to aid in an initial global search for the fold, while the structure module further refines the prediction. Consistent with this, in the absence of an MSA, providing a good template rescues structure prediction performance (Roney & Ovchinnikov, 2022). To test whether good templates can compensate for the lack of an MSA in protein‐peptide docking, we focused on AF2‐Multimer and AF3, because Boltz‐1 and Chai‐1 did not support custom templates at the time of this study. For AF3, but not AF2‐Multimer, using native templates for both protein and peptide (taken from the experimental structure of the complex), without any protein or peptide MSA, modestly yet significantly outperformed using unpaired MSAs without any templates (Figure S16a). We found that providing native templates in addition to protein MSAs boosted structure prediction accuracy for the protein and overall docking accuracy (Figure S16a).

To determine whether the protein MSA primarily supports prediction of the protein structure, or if the evolutionary statistics in the MSA also inform docking, we compared predictions made with the protein MSA to predictions for which only a template was provided for the protein; the peptide was provided as a sequence with no template or MSA in both. AF2‐Multimer was sensitive to this difference, performing significantly worse when provided with only the protein template, even though the protein structure was predicted more accurately (Figure S17a). AF3 performed similarly with both input conditions. We repeated this experiment for the peptide MSA, using the protein MSA with no protein template. For the subset of complexes where the peptide MSA had at least 50 sequences, we used either the peptide MSA or the native peptide structure as a template for the input. AF2‐Multimer and AF3 performed slightly better when provided the peptide template (Figure S17b). Most DockQ improvements were small, indicating local structural refinements. These results show that for AF2‐Multimer, the evolutionary information available in the protein MSA cannot be fully replaced by structural information. In contrast, the evolutionary information in the peptide MSA, which is typically lower due to shallower MSAs, can be replaced and even improved upon with structural information. To validate this finding further for the protein MSA, we repeated the test with the masked peptide sequences and, as expected, found that AF2‐Multimer performed significantly worse when provided with the protein template than with the protein MSA, as assessed by RMSD_map_ (Figure S17c). This highlights the importance of the protein MSA for AF2 binding site identification.

**FIGURE 6:**
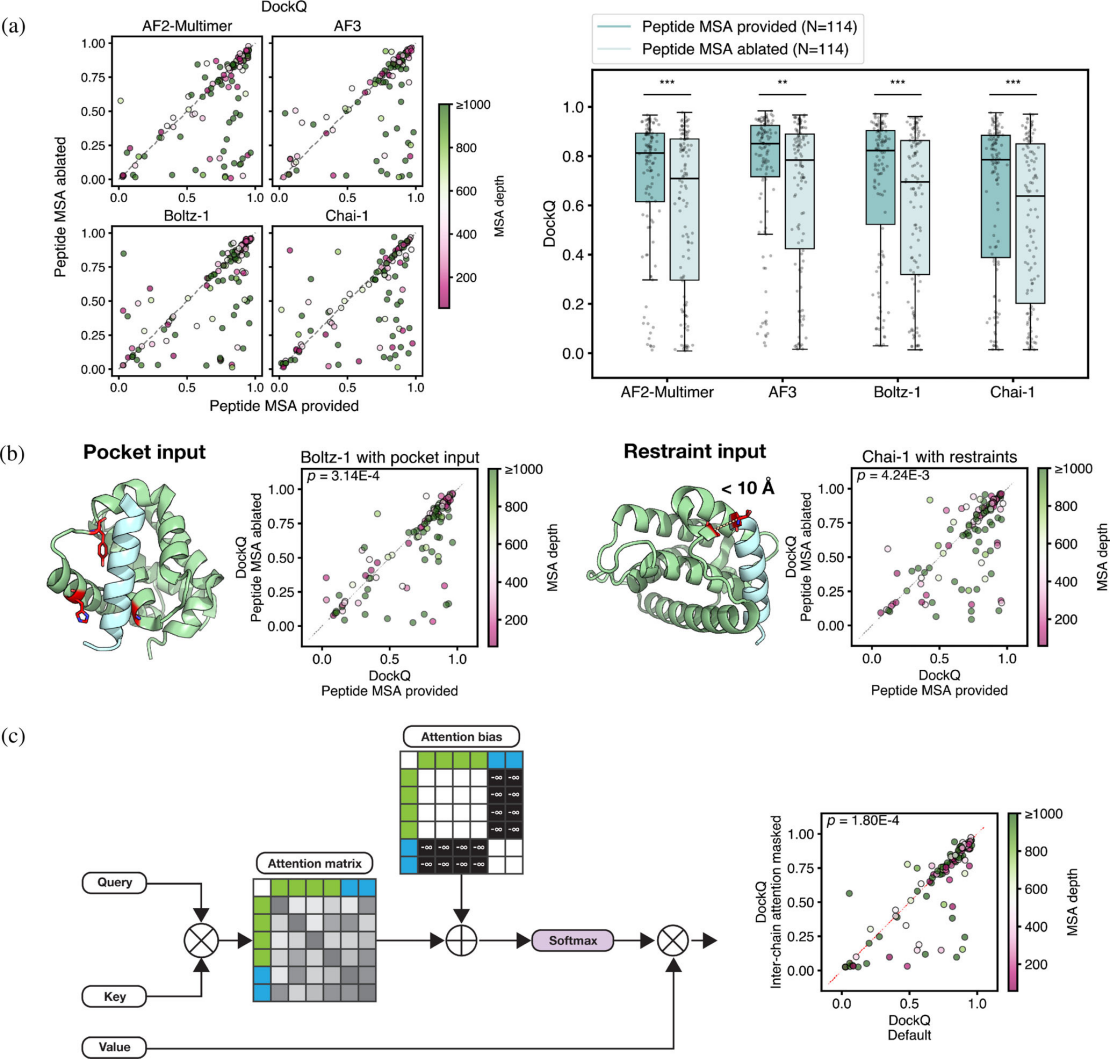
Peptide MSAs boost performance. (a) Comparison of DockQ between using the unpaired MSA versus the peptide‐ablated MSA for complexes where the peptide MSA had at least 50 sequences (*p < 0.05, **p < 0.01, ***p < 0.001). (b) Left: Example of Boltz‐1 prediction with pocket input, where the three residues in red were designated as pocket residues. The plot shows DockQ scores using the unpaired MSA versus peptide‐ablated MSA when the Boltz‐1 pocket input was provided; Right: Example of Chai‐1 prediction in which a 10 Å distance restraint was applied to the indicated inter‐residue distance. The plot shows DockQ scores using the unpaired MSA versus peptide‐ablated MSA when the Chai‐1 restraint input was provided. (c) Left: Procedure for masking interchain attention in the Evoformer module of AF2‐Multimer; Right: DockQ for predictions made with inter‐chain attention unmasked (horizontal axis) versus inter‐chain attention masked (vertical axis). All *p*‐values are from a Wilcoxon signed‐rank test comparing the two distributions.

In most use cases, the bound peptide conformation is not known and can't be provided as a template. Thus, we dissected how the peptide MSA is used. We tested whether the peptide MSA contributes to docking performance by removing it for complexes where the peptide MSA (made with default methods) had at least 50 sequences. Interestingly, for most complexes, ablating the peptide MSA had little impact (Figure 6a). Overall, peptide MSA ablation slightly reduced performance, with median DockQ dropping from 0.79–0.85 to 0.64–0.78 across the models. AF2‐Multimer was the most sensitive to this ablation. Of the complexes for which the original prediction had medium DockQ, 74% maintained medium DockQ after peptide MSA ablation. AF3 was the least sensitive, with 83% of medium predictions maintaining medium DockQ after the ablation. For cases where peptide MSA ablation decreased accuracy, it impacted both binding site selection and peptide conformation, as illustrated by examples where DockQ dropped from excellent to medium (i.e., the peptide conformation was predicted less accurately) and when DockQ dropped from excellent or medium to poor (i.e., the binding site was predicted incorrectly). Ablating the peptide MSA significantly decreased performance for complexes in the pre‐training date cutoff set. The post‐training date cutoff set did not contain enough examples to support this test (Figure S13).

To assess the extent to which the peptide MSAs inform the peptide binding conformation, beyond binding site prediction, we made predictions where the binding site was provided to the model. We focused on Boltz‐1 and Chai‐1 for this experiment because these models provided the option to specify binding‐site residues or distance restraints. For Boltz‐1, three binding‐site residues from the protein were designated as part of the “pocket” (Figure 6b, left). For Chai‐1, we included a single distance restraint between a binding‐site residue and a peptide residue (≤10 Å) (Figure 6b, right). Providing constraints improved accuracy (Figure S18). But when constraints were provided, ablating the peptide MSA (for peptide MSAs with at least 50 sequences) still resulted in a decrease in accuracy, demonstrating that the peptide MSA contributes to the prediction of the peptide binding conformation (Figure 6b).

To understand how AF2‐Multimer processes unpaired protein and peptide MSA information together in the Evoformer module, we masked row‐ and column‐wise self‐attention between rows and columns corresponding to different chains in the MSA (Figure 6c, left). In this masked attention regime, the model may leverage intra‐chain evolutionary information and develop an understanding of each chain individually, but cannot process the two MSAs together in the Evoformer module (Methods, “Inter‐chain attention masking”). We saw significantly lower performance with this masking protocol on some of the complexes where the peptide MSA had at least 50 sequences (Figure 6c, right). Although coevolutionary statistics cannot be directly extracted, as is possible for a paired MSA, the Evoformer is nonetheless able to learn from inter‐chain attention on an unpaired MSA.

### 2.5 Explaining successful AF3 predictions of novel complexes

All models performed very well on complexes with similar structures in the training data, suggesting that successful prediction may be achieved using a kind of homology modeling. Nevertheless, some test cases that lacked similarity to training‐set complexes were still accurately predicted. We manually investigated six complexes that AF3 predicted with DockQ >0.49 despite lacking binding‐site matches in the training set: 8BRH_AB, 8S6O_DH, 8FG6_BA, 8HDJ_BA, 8TEE_AC, and 9G6Z_AB (Chen et al., 2023; Devan et al., 2024; PDB, 2023b; PDB, 2024c; PDB, 2024d; PDB, 2024e; PDB, 2024f; Podvalnaya et al., 2023; Sahtoe et al., 2024; Sidibé et al., 2024; Vogel et al., 2024). For 8BRH_AB, the structure of yeast She4 with a Myo4 peptide, AF3 was successful when the protein MSA was provided, but unsuccessful when the She4 template was provided with no protein or peptide MSA (Figure S19a). We concluded that there is some essential evolutionary information present in the protein MSA and note that the native binding site is more highly conserved than the rest of the protein, which could facilitate binding‐site identification. In contrast, for 8S6O_DH, the structure of the MLLE3 domain of Rrm4 bound to a Upa1 PAM2L2 motif, AF3 was able to dock the peptide when provided the MLLE3 template and no protein or peptide MSA (Figure S19b, upper left). Since the native binding site is a hydrophobic cleft and the peptide has two aromatic residues, we hypothesized that AF3 chose a pose that buries hydrophobic residues (Figure S19b, upper right). Supporting this, when phenylalanine and tyrosine in the peptide DE**F**I**Y**P were mutated to alanine, AF3 did not recover the native peptide backbone conformation but still chose the native binding site (Figure S19b, lower left). When all residues in the Rrm4 binding site were mutated to alanine, AF3 no longer predicted that site (Figure S19b, lower right). For the remaining four examples, we attribute the success of AF3 to the identification of common secondary structure packing motifs across the interface. For example, for the designed complex 8FG6_BA, and for the structure of RsgI2 of *Clostridium thermocellum* (8HDJ_BA), AF3 consistently captured the β‐sheet formed by the peptide at the correct binding site (Figure S19c, first and second row). For 8TEE_AC, the structure of Kindlin2 in complex with a peptide from integrin‐β1, the native structure also features a β‐sheet interaction. When we mutated the 12 interfacial peptide residues of integrin‐β1 to alanines, which have high helical propensity, AF3 predicted a helical conformation for the peptide and repositioned it to pack against an α‐helix in the protein (Figure S19c, third row). Here, the predicted secondary structure of the peptide may have influenced the binding mode. Finally, for 9G6Z_AB, the structure of *C. elegans* TOFU‐6 eTUDOR TOFU‐1 peptide complex, AF3 predicted the peptide to adopt an α‐helix at the native binding site, which consists of several small helices (Figure S19c, last row).

## 3 DISCUSSION

In this study, we evaluated the performance of AF2‐Multimer, AF3, Boltz‐1, and Chai‐1 on the task of protein‐peptide complex structure prediction. Most predictions identified the correct binding site, with DockQ >0.49, and many were highly accurate. AF3 slightly outperformed Boltz‐1 and Chai‐1, potentially due to a larger distilled training data set and increased ability to memorize information in the training set, as indicated by the sensitivity of the performance of AF3 to training set similarity (Chai Discovery team et al., 2024; Wohlwend et al., 2024). Data leakage into test sets constructed based on a training‐date cutoff is expected for the PDB, where many proteins and protein complexes are represented with hundreds of related, although non‐identical, structures. To address this, we added another test for training‐test set overlap based on complex structural similarity. For all models, binding sites that were more represented in the training set were predicted more accurately. In some cases, the training data may have led the model to use an incorrect but commonly seen binding site (Figure 3). Among predictions with DockQ <0.23, 80%–86% involved complexes with one or more characteristics correlated with poorer performance, including irregular secondary structure and structural novelty (Figure S20).

Our findings align with a previous benchmark of AF2‐Multimer reporting that the model struggles with predicting PROTAC‐mediated protein–protein interfaces (Pereira et al., 2024), which tend to involve proteins that do not interact in natural systems and are thus unlikely to be represented in training sets. Additionally, our observations align with AF3, Boltz‐1, and Chai‐1 benchmarks for small‐molecule docking, where ligands with abundant data in the PDB show better prediction performance (Škrinjar et al., 2025, Masters et al., 2025). It is still common practice to use homology splits based on single chains and/or temporal splits for training and evaluation. The significant differences in prediction quality for pre‐ versus post‐training examples, and for examples with versus without binding‐site matches in the training data (Figure 2), highlight the need for non‐redundant evaluation sets to estimate performance on novel tasks. In our dataset, however, we observed very few cases with zero binding‐site matches, underscoring how limited the available pool of unseen structures is for benchmarking. Bias for previously observed complexes raises a concern for generalization to prediction tasks of increasing complexity, for example, for protein–protein interactions and biomolecular assemblies that are not well‐represented in the PDB. Designing novel interactions and targeting new binding sites when designing peptide binders also require this type of extrapolation. In our evaluations, ligands or post‐translational modifications (PTMs) were not modeled, to enable fair comparison with AF2‐Multimer, which cannot model these features. Understanding the impact of such groups on the prediction accuracy of generalized biomolecular prediction models (such as AF3, Boltz‐1, and Chai‐1) will be an important direction for future work.

Although previously seen structures were predicted more accurately, we found cases where novel interactions were predicted correctly. Additionally, there were cases of PDB structures present in the training set that the models failed to predict. These surprising examples led us to investigate the role of the MSA input. First, we found that models do not rely on inter‐chain coevolutionary signal (Figure 4). This is in contrast with the predominant theory that AF2‐Multimer folds protein–protein complexes based on coevolutionary information between chains, which led the developers of AF2 and other models to pair sequences by species in the MSA construction process. Further, for protein–protein interactions, Bryant et al. found that a paired + unpaired MSA resulted in the best predictions for AF2 (trained on single‐chain proteins) and that the number of sequences in the paired MSA had a strong influence on predictions (Bryant et al., 2022). These observations did not clarify whether the pairing in the MSA or the increased depth of the MSAs is responsible for the boost in prediction quality. In our experiments, we found weak evidence that models have learned to incorporate coevolution, as defined by patterns detectable after sequence pairing.

Second, across the models, we found that the peptide MSA improves 17%–26% of complex predictions, influencing both binding‐site and binding‐pose prediction. For AF2‐Multimer, we found evidence indicating that inter‐chain interactions are implicitly extracted from an MSA through the Evoformer mechanism of interleaving row‐ and column‐wise attention without requiring sequence pairing. Because the MSA modules of AF3 and related models do not use column‐wise attention, we leave the interrogation of how inter‐chain statistics are processed in these models to future work.

Third, in the absence of a paired MSA, we found that the protein MSA is not only used to determine the structure of the protein but often contains sufficient information for the prediction of binding sites, as illustrated by our tests in which the peptide sequence was masked (Figure 5). AF2 has previously been adapted for predicting small‐molecule ligand binding sites on target proteins (Gazizov et al., 2023). Conserved positions on the protein do not, alone, explain the choice of binding site (Figure S14), though previous work that involved tuning the AF2 MSA profile for improving predictions suggests that statistics of residue frequencies provided to the model play an important role (Bryant & Noé, 2023; Fadini et al., 2025). Our findings suggest that docking specificity is a challenge, particularly among highly similar peptides or proteins, because models will often dock peptides without a peptide MSA or even a peptide sequence. Several studies have benchmarked AF2 and AF2‐Multimer for binder or short linear motif (SLiM) discovery; these have found moderate specificity (Bret et al., 2024; Lee et al., 2024; Savinov et al., 2025; Tsaban et al., 2022). For example, Lee et al. found that AF2‐Multimer discriminated poorly between binding SLiMs and SLiMs with one mutation introduced in a conserved motif position (Lee et al., 2024).

Close inspection of the few highly successful novel predictions by AF3 showed that most recognize common secondary structure element interactions at the interface. We found one example where the evolutionary information in the protein MSA was critical for docking success (Figure S19a). While binding site conservation and hydrophobicity are possible explanations for the accurate modeling of two complexes, these were not strongly predictive features in our benchmark overall. Our results demonstrate that peptide docking success cannot be explained using simple MSA‐based statistics alone, since models have learned to use sources of information beyond covariation. Future research on understanding how these models make their predictions should take inspiration from studies explaining the behavior of protein language models, such as ESM‐2 (Adams et al., 2025; Simon & Zou, 2025; Vinod et al., 2025; Zhang et al., 2024). For example, Zhang et al. found that ESM‐2 predicts structures by pairing local structural motifs rather than memorizing domains or folds (Zhang et al., 2024). Similar efforts for MSA‐based sequence‐to‐structure models will continue to advance our understanding of how these widely used methods make predictions, particularly as methods like MSA subsampling and tuning have brought new capabilities to computational structure prediction (Bryant & Noé, 2023; del Alamo et al., 2022; Fadini et al., 2025; Wayment‐Steele et al., 2024).

## AUTHOR CONTRIBUTIONS

**Lindsey Guan:** Conceptualization; investigation; writing – original draft; software; data curation; writing – review and editing. **Amy E. Keating:** Conceptualization; writing – original draft; supervision; writing – review and editing.

## Supporting information


**Data S1:** Supporting Information.

## Data Availability

The data that support the findings of this study are openly available in Structural data at https://zenodo.org/records/16929570?token=eyJhbGciOiJIUzUxMiJ9.eyJpZCI6IjcxNDk3ZTRjLTllZjgtNDA4Ny05YjAzLWFiMDI1ZTQxMWY0NiIsImRhdGEiOnt9LCJyYW5kb20iOiI3NDVkYjc5ZTdjYTZjZmQ0NDA3OGNiOGQ0MTg4ZDAxYiJ9.acjq6‐EXexeqEhAjc7IXDRcTfKI6LTchDAgFHAzSFGEtY9No8y9OvvZLF1Q.

## References

[pro70331-bib-0001] Abramson J , Adler J , Dunger J , Evans R , Green T , Pritzel A , et al. Accurate structure prediction of biomolecular interactions with AlphaFold 3. Nature. 2024;630:493–500. 38718835 10.1038/s41586-024-07487-w PMC11168924

[pro70331-bib-0002] Adams E , Bai L , Lee M , Yu Y , AlQuraishi M . From Mechanistic Interpretability to Mechanistic Biology: Training, Evaluating, and Interpreting Sparse Autoencoders on Protein Language Models. *bioRxiv* p. 2025.02.06.636901. 2025.

[pro70331-bib-0003] Aguilar F , Yu S , Grant RA , Swanson S , Ghose D , Su BG , et al. Peptides from human BNIP5 and PXT1 and non‐native binders of pro‐apoptotic BAK can directly activate or inhibit BAK‐mediated membrane permeabilization. Structure. 2023;31:265–281.e7. 36706751 10.1016/j.str.2023.01.001 PMC9992319

[pro70331-bib-0004] Anishchenko I , Pellock SJ , Chidyausiku TM , Ramelot TA , Ovchinnikov S , Hao J , et al. De novo protein design by deep network hallucination. Nature. 2021;600:547–552. 34853475 10.1038/s41586-021-04184-w PMC9293396

[pro70331-bib-0005] Basu S , Wallner B . DockQ: a quality measure for protein‐protein docking models. PLoS One. 2016;11:e0161879. 27560519 10.1371/journal.pone.0161879 PMC4999177

[pro70331-bib-0006] Bedding MJ , Franck C , Johansen‐Leete J , Aggarwal A , Maxwell JWC , Patel K , et al. Discovery of high affinity cyclic peptide ligands for human ACE2 with SARS‐CoV‐2 entry inhibitory activity. ACS Chem Biol. 2024;19:141–152. 38085789 10.1021/acschembio.3c00568

[pro70331-bib-0007] Bennett NR , Coventry B , Goreshnik I , Huang B , Allen A , Vafeados D , et al. Improving de novo protein binder design with deep learning. Nat Commun. 2023;14:2625. 37149653 10.1038/s41467-023-38328-5 PMC10163288

[pro70331-bib-0008] Bret H , Gao J , Zea DJ , Andreani J , Guerois R . From interaction networks to interfaces, scanning intrinsically disordered regions using AlphaFold2. Nat Commun. 2024;15:597. 38238291 10.1038/s41467-023-44288-7 PMC10796318

[pro70331-bib-0009] Bryant P , Noé F . Improved protein complex prediction with AlphaFold‐multimer by denoising the MSA profile. *bioRxiv* p. 2023.07.04.547638. 2023. 10.1371/journal.pcbi.1012253 PMC11302914 39052676

[pro70331-bib-0010] Bryant P , Pozzati G , Elofsson A . Improved prediction of protein‐protein interactions using AlphaFold2. Nat Commun. 2022;13:1265. 35273146 10.1038/s41467-022-28865-w PMC8913741

[pro70331-bib-0011] Capra JA , Singh M . Predicting functionally important residues from sequence conservation. Bioinformatics. 2007;23:1875–1882. 17519246 10.1093/bioinformatics/btm270

[pro70331-bib-0012] Chai Discovery team , Boitreaud J , Dent J , McPartlon M , Meier J , Reis V , et al. Chai‐1: Decoding the molecular interactions of life. *bioRxiv* p. 2024.10.10.615955. 2024.

[pro70331-bib-0013] Chang C‐W , Couñago RLM , Williams SJ , Bodén M , Kobe B . Crystal structure of rice importin‐a and structural basis of its interaction with plant‐specific nuclear localization signals. Plant Cell. 2012;24:5074–5088. 23250448 10.1105/tpc.112.104422 PMC3556976

[pro70331-bib-0014] Chen C , Dong S , Yu Z , Qiao Y , Li J , Ding X , et al. Essential autoproteolysis of bacterial anti‐σ factor RsgI for transmembrane signal transduction. Sci Adv. 2023;9:eadg4846. 37418529 10.1126/sciadv.adg4846 PMC10328401

[pro70331-bib-0015] Chica C , Labarga A , Gould CM , López R , Gibson TJ . A tree‐based conservation scoring method for short linear motifs in multiple alignments of protein sequences. BMC Bioinformatics. 2008;9:229. 18460207 10.1186/1471-2105-9-229 PMC2396637

[pro70331-bib-0016] Dana JM , Gutmanas A , Tyagi N , Qi G , O'Donovan C , Martin M , et al. SIFTS: updated structure integration with function, taxonomy and sequences resource allows 40‐fold increase in coverage of structure‐based annotations for proteins. Nucleic Acids Res. 2019;47:D482–D489. 30445541 10.1093/nar/gky1114 PMC6324003

[pro70331-bib-0017] Davey NE , Cowan JL , Shields DC , Gibson TJ , Coldwell MJ , Edwards RJ . SLiMPrints: conservation‐based discovery of functional motif fingerprints in intrinsically disordered protein regions. Nucleic Acids Res. 2012;40:10628–10641. 22977176 10.1093/nar/gks854 PMC3510515

[pro70331-bib-0018] del Alamo D , Sala D , Mchaourab HS , Meiler J . Sampling alternative conformational states of transporters and receptors with AlphaFold2. eLife. 2022;11:e75751. 35238773 10.7554/eLife.75751 PMC9023059

[pro70331-bib-0019] Devan S‐K , Shanmugasundaram S , Müntjes K , Postma J , Smits SHJ , Altegoer F , et al. Deciphering the RNA‐binding protein network during endosomal mRNA transport. Proc Natl Acad Sci. 2024;121(46):e2404091121. 39499630 10.1073/pnas.2404091121 PMC11572963

[pro70331-bib-0020] Evans R , O'Neill M , Pritzel A , Antropova N , Senior A , Green T , et al. Protein complex prediction with AlphaFold‐Multimer. *bioRxiv* p. 2021.10.04.463034. 2022.

[pro70331-bib-0021] Fadini A , Li M , McCoy AJ , Terwilliger TC , Read RJ , Hekstra D , et al. AlphaFold as a Prior: Experimental Structure Determination Conditioned on a Pretrained Neural Network. *bioRxiv* p. 2025.02.18.638828. 2025. 10.1038/s41592-026-03047-4 PMC13124595 41922571

[pro70331-bib-0022] Gazizov A , Lian A , Goverde C , Ovchinnikov S , Polizzi NF . AF2BIND: Predicting ligand‐binding sites using the pair representation of AlphaFold2. *bioRxiv* p. 2023.10.15.562410. 2023. 10.1038/s41592-026-03011-2 PMC12982129 41814061

[pro70331-bib-0023] Gloor GB , Martin LC , Wahl LM , Dunn SD . Mutual information in protein multiple sequence alignments reveals two classes of coevolving positions. Biochemistry. 2005;44:7156–7165. 15882054 10.1021/bi050293e

[pro70331-bib-0024] Goudy OJ , Nallathambi A , Kinjo T , Randolph NZ , Kuhlman B . In silico evolution of autoinhibitory domains for a PD‐L1 antagonist using deep learning models. Proc Natl Acad Sci USA. 2023;120:e2307371120. 38032933 10.1073/pnas.2307371120 PMC10710080

[pro70331-bib-0025] Goverde CA , Wolf B , Khakzad H , Rosset S , Correia BE . De novo protein design by inversion of the AlphaFold structure prediction network. Protein Sci. 2023;32:e4653. 37165539 10.1002/pro.4653 PMC10204179

[pro70331-bib-0026] Green AG , Elhabashy H , Brock KP , Maddamsetti R , Kohlbacher O , Marks DS . Large‐scale discovery of protein interactions at residue resolution using co‐evolution calculated from genomic sequences. Nat Commun. 2021;12:1396. 33654096 10.1038/s41467-021-21636-z PMC7925567

[pro70331-bib-0027] Guo Q , Liao S , Kwiatkowski S , Tomaka W , Yu H , Wu G , et al. Structural insights into SETD3‐mediated histidine methylation on β‐actin. elife. 2019;8:e43676. 30785395 10.7554/eLife.43676 PMC6400499

[pro70331-bib-0028] Janin J . Assessing predictions of protein‐protein interaction: the CAPRI experiment. Protein Sci. 2005;14:278–283. 15659362 10.1110/ps.041081905 PMC2253420

[pro70331-bib-0029] Johansson‐Åkhe I , Wallner B . Improving peptide‐protein docking with AlphaFold‐Multimer using forced sampling. Front Bioinform. 2022;2:959160. 36304330 10.3389/fbinf.2022.959160 PMC9580857

[pro70331-bib-0030] Joosten RP , te Beek TA , Krieger E , Hekkelman ML , Hooft RW , Schneider R , et al. A series of PDB related databases for everyday needs. Nucleic Acids Res. 2011;39:D411–D419. 21071423 10.1093/nar/gkq1105 PMC3013697

[pro70331-bib-0031] Jumper J , Evans R , Pritzel A , Green T , Figurnov M , Ronneberger O , et al. Highly accurate protein structure prediction with AlphaFold. Nature. 2021;596:583–589. 34265844 10.1038/s41586-021-03819-2 PMC8371605

[pro70331-bib-0032] Kabsch W , Sander C . Dictionary of protein secondary structure: pattern recognition of hydrogen‐bonded and geometrical features. Biopolymers. 1983;22:2577–2637. 6667333 10.1002/bip.360221211

[pro70331-bib-0033] Kim K , Min J , Kirby TW , Gabel SA , Pedersen LC , London RE . Ligand binding characteristics of the Ku80 von Willebrand domain. DNA Repair. 2020;85:102739. 31733588 10.1016/j.dnarep.2019.102739 PMC7495496

[pro70331-bib-0034] Ko J , Lee J . Can AlphaFold2 predict protein‐peptide complex structures accurately? *bioRxiv* p. 2021.07.27.453972. 2021.

[pro70331-bib-0035] Kosugi S , Hasebe M , Matsumura N , Takashima H , Miyamoto‐Sato E , Tomita M , et al. Six classes of nuclear localization signals specific to different binding grooves of importin α*. J Biol Chem. 2009;284:478–485. 19001369 10.1074/jbc.M807017200

[pro70331-bib-0036] Ku B , Woo J‐S , Liang C , Lee K‐H , Hong H‐S , Kim K‐S , et al. Structural and biochemical bases for the inhibition of autophagy and apoptosis by viral BCL‐2 of murine γ‐herpesvirus 68. PLoS Pathog. 2008;4:e25. 18248095 10.1371/journal.ppat.0040025 PMC2222952

[pro70331-bib-0037] Lee CY , Hubrich D , Varga JK , Schäfer C , Welzel M , Schumbera E , et al. Systematic discovery of protein interaction interfaces using AlphaFold and experimental validation. Mol Syst Biol. 2024;20:75–97. 38225382 10.1038/s44320-023-00005-6 PMC10883280

[pro70331-bib-0038] Li S , Li T , Xu Y , Zhang Q , Zhang W , Che S , et al. Structural insights into YfiR sequestering by YfiB in Pseudomonas aeruginosa PAO1. Sci Rep. 2015;5:16915. 26593397 10.1038/srep16915 PMC4655355

[pro70331-bib-0039] Lin Z , Akin H , Rao R , Hie B , Zhu Z , Lu W , et al. Evolutionary‐scale prediction of atomic‐level protein structure with a language model. Science. 2023;379:1123–1130. 36927031 10.1126/science.ade2574

[pro70331-bib-0040] Martin LC , Gloor GB , Dunn SD , Wahl LM . Using information theory to search for co‐evolving residues in proteins. Bioinformatics. 2005;21:4116–4124. 16159918 10.1093/bioinformatics/bti671

[pro70331-bib-0041] Martins PM , Santos LH , Mariano D , Queiroz FC , Bastos LL , Gomes I d S , et al. Propedia: a database for protein–peptide identification based on a hybrid clustering algorithm. BMC Bioinformatics. 2021;22:1. 33388027 10.1186/s12859-020-03881-z PMC7776311

[pro70331-bib-0084] Masters MR , Mahmoud AH , Lill MA . Investigating whether deep learning models for co‐folding learn the physics of protein‐ligand interactions. Nat Commun.2025;16:8854. 41053181 10.1038/s41467-025-63947-5 PMC12501370

[pro70331-bib-0042] Mirabello C , Wallner B . DockQ v2: improved automatic quality measure for protein multimers, nucleic acids, and small molecules. Bioinformatics. 2024;40:btae586. 39348158 10.1093/bioinformatics/btae586 PMC11467047

[pro70331-bib-0044] Nemoz C , Legrand P , Ropars V , Charbonnier JB . Complex of APLF factor and Ku heterodimer bound to DNA. 2018b.

[pro70331-bib-0043] Nemoz C , Legrand P , Ropars V , Charbonnier JB . Complex of XLF and heterodimer Ku bound to DNA. 2018a.

[pro70331-bib-0045] Nemoz C , Ropars V , Frit P , Gontier A , Drevet P , Yu J , et al. XLF and APLF bind Ku80 at two remote sites to ensure DNA repair by non‐homologous end joining. Nat Struct Mol Biol. 2018;25:971–980. 30291363 10.1038/s41594-018-0133-6 PMC6234012

[pro70331-bib-0055] PDB . ACE2‐peptide 1 complex. 2024a.

[pro70331-bib-0060] PDB . C. elegans TOFU‐6 eTUDOR TOFU‐1 peptide complex. 2024f.

[pro70331-bib-0057] PDB . Co‐crystal structure of She4 with Myo4 peptide. 2024c.

[pro70331-bib-0049] PDB . Crystal structure of a bacterial signalling complex. 2016.

[pro70331-bib-0059] PDB . Crystal structure of Kindlin2 in complex with K794Q mutated beta1 integrin. 2024e.

[pro70331-bib-0046] PDB . Crystal Structure of M11, the BCL‐2 Homolog of Murine Gamma‐herpesvirus 68, Complexed with Mouse Beclin1 (residues 106–124). 2008.

[pro70331-bib-0052] PDB . Crystal structure of RCC1‐Like domain 2 of ubiquitin ligase HERC2 in complex with DXDKDED motif of dedicator of cytokinesis protein 10. 2022.

[pro70331-bib-0053] PDB . Human BAK in complex with the dF3 peptide. 2023a.

[pro70331-bib-0054] PDB . Periplasmic domain of RsgI2 of Clostridium thermocellum. 2023b.

[pro70331-bib-0048] PDB . rImp_alpha_a89nls. 2013.

[pro70331-bib-0056] PDB . Structure of MLLE domain of Pab1 in complex with PAM2 of Upa1. 2024b.

[pro70331-bib-0058] PDB . Structure of MLLE3 domain of Rrm4 in complex with PAM2L2 of Upa1. 2024d.

[pro70331-bib-0051] PDB . Structure of SETD3 bound to SAH and unmodified actin. 2019b.

[pro70331-bib-0047] PDB . The crystal structure of aspergilloglutamic peptidase from Aspergillus niger. 2012.

[pro70331-bib-0050] PDB . The crystal structure of muPAin‐1‐IG‐2 in complex with muPA‐SPD at pH8.5. 2019a.

[pro70331-bib-0061] Pereira GP , Gouzien C , Souza PCT , Martin J . Challenges in predicting PROTAC‐mediated protein–protein interfaces with AlphaFold reveal a general limitation on small interfaces. Bioinformatics Advances. 2025;5:vbaf056. 40144455 10.1093/bioadv/vbaf056 PMC11938821

[pro70331-bib-0062] Pereira J , Simpkin AJ , Hartmann MD , Rigden DJ , Keegan RM , Lupas AN . High‐accuracy protein structure prediction in CASP14. Proteins. 2021;89:1687–1699. 34218458 10.1002/prot.26171

[pro70331-bib-0063] Petsalaki E , Russell RB . Peptide‐mediated interactions in biological systems: new discoveries and applications. Protein Technol/Systems Biol. 2008;19:344–350. 10.1016/j.copbio.2008.06.004 18602004

[pro70331-bib-0064] Podvalnaya N , Bronkhorst AW , Lichtenberger R , Hellmann S , Nischwitz E , Falk T , et al. piRNA processing by a trimeric Schlafen‐domain nuclease. Nature. 2023;622:402–409. 37758951 10.1038/s41586-023-06588-2 PMC10567574

[pro70331-bib-0065] Roney JP , Ovchinnikov S . State‐of‐the‐art estimation of protein model accuracy using AlphaFold. Phys Rev Lett. 2022;129:238101. 36563190 10.1103/PhysRevLett.129.238101 PMC12178128

[pro70331-bib-0066] Sahtoe DD , Andrzejewska EA , Han HL , Rennella E , Schneider MM , Meisl G , et al. Design of amyloidogenic peptide traps. Nat Chem Biol. 2024;20:981–990. 38503834 10.1038/s41589-024-01578-5 PMC11288891

[pro70331-bib-0067] Sasaki H , Kubota K , Lee WC , Ohtsuka J , Kojima M , Iwata S , et al. The crystal structure of an intermediate dimer of aspergilloglutamic peptidase that mimics the enzyme‐activation product complex produced upon autoproteolysis. J Biochem. 2012;152:45–52. 22569035 10.1093/jb/mvs050

[pro70331-bib-0068] Savinov A , Swanson S , Keating AE , Li G‐W . High‐throughput discovery of inhibitory protein fragments with AlphaFold. Proc Natl Acad Sci USA. 2025;122:e2322412122. 39899719 10.1073/pnas.2322412122 PMC11831152

[pro70331-bib-0069] Sidibé A , Mykuliak VV , Zhang P , Hytönen VP , Wu J , Wehrle‐Haller B . Acetyl‐NPKY of integrin‐β1 binds KINDLIN2 to control endothelial cell proliferation and junctional integrity. iScience. 2024;27:110129. 38904068 10.1016/j.isci.2024.110129 PMC11187247

[pro70331-bib-0070] Simon E , Zou J . InterPLM: Discovering Interpretable Features in Protein Language Models via Sparse Autoencoders. Nat Methods. 2025. 10.1038/s41592-025-02836-7 41023434

[pro70331-bib-0071] Škrinjar P , Eberhardt J , Durairaj J , Schwede T . Have protein‐ligand co‐folding methods moved beyond memorisation? *bioRxiv* p. 2025.02.03.636309. 2025.

[pro70331-bib-0072] Tillier ERM , Lui TWH . Using multiple interdependency to separate functional from phylogenetic correlations in protein alignments. Bioinformatics. 2003;19:750–755. 12691987 10.1093/bioinformatics/btg072

[pro70331-bib-0073] Tsaban T , Varga JK , Avraham O , Ben‐Aharon Z , Khramushin A , Schueler‐Furman O . Harnessing protein folding neural networks for peptide–protein docking. Nat Commun. 2022;13:176. 35013344 10.1038/s41467-021-27838-9 PMC8748686

[pro70331-bib-0074] Villa F , Goebel J , Rafiqi FH , Deak M , Thastrup J , Alessi DR , et al. Structural insights into the recognition of substrates and activators by the OSR1 kinase. EMBO Rep. 2007;8(9):839–845. 17721439 10.1038/sj.embor.7401048 PMC1973955

[pro70331-bib-0075] Vinod R , Amini AP , Crawford L , Yang KK . Trainable subnetworks reveal insights into structure knowledge organization in protein language models. *bioRxiv* p. 2025.05.29.656902. 2025. 10.1371/journal.pcbi.1013925 PMC12928587 41662462

[pro70331-bib-0076] Vogel A , Arnese R , Gudino Carrillo RM , Sehr D , Deszcz L , Bylicki A , et al. UNC‐45 assisted myosin folding depends on a conserved FX3HY motif implicated in freeman Sheldon syndrome. Nat Commun. 2024;15:6272. 39054317 10.1038/s41467-024-50442-6 PMC11272940

[pro70331-bib-0077] Wallner B . AFsample: improving multimer prediction with AlphaFold using massive sampling. Bioinformatics. 2023;39:btad573. 37713472 10.1093/bioinformatics/btad573 PMC10534052

[pro70331-bib-0078] Wang D , Yang Y , Jiang L , Wang Y , Li J , Andreasen PA , et al. Suppression of tumor growth and metastases by targeted intervention in Urokinase activity with cyclic peptides. J Med Chem. 2019;62:2172–2183. 30707839 10.1021/acs.jmedchem.8b01908

[pro70331-bib-0079] Wayment‐Steele HK , Ojoawo A , Otten R , Apitz JM , Pitsawong W , Hömberger M , et al. Predicting multiple conformations via sequence clustering and AlphaFold2. Nature. 2024;625:832–839. 37956700 10.1038/s41586-023-06832-9 PMC10808063

[pro70331-bib-0080] Wohlwend J , Corso G , Passaro S , Reveiz M , Leidal K , Swiderski W , et al. Boltz‐1 Democratizing Biomolecular Interaction Modeling. *bioRxiv* p. 2024.11.19.624167. 2024.

[pro70331-bib-0081] Wu R , Ding F , Wang R , Shen R , Zhang X , Luo S , et al. High‐resolution de novo structure prediction from primary sequence. *bioRxiv* p. 2022.07.21.500999. 2022.

[pro70331-bib-0082] Zhang Z , Wayment‐Steele HK , Brixi G , Wang H , Kern D , Ovchinnikov S . Protein language models learn evolutionary statistics of interacting sequence motifs. Proc Natl Acad Sci USA. 2024;121:e2406285121. 39467119 10.1073/pnas.2406285121 PMC11551344

[pro70331-bib-0083] Zhu W , Shenoy A , Kundrotas P , Elofsson A . Evaluation of AlphaFold‐Multimer prediction on multi‐chain protein complexes. Bioinformatics. 2023;39:btad424. 37405868 10.1093/bioinformatics/btad424 PMC10348836

