# Supplementary material for: Training bias and sequence alignments shape protein–peptide docking by AlphaFold and related methods
Source: Protein Sci. 2025 Oct 13;34(11):e70331. doi: 10.1002/pro.70331 (PMC12518507; doi:10.1002/pro.70331)
Supplement: Supplementary file 1 — Data S1: Supporting Information. [file PRO-34-e70331-s001.pdf]

Supplementary Information for

Training Bias and Sequence Alignments Shape Protein–Peptide Docking by AlphaFold and Related Methods

Lindsey Guan and Amy E. Keating*

Protein Science, 2025

*Corresponding author. Email: keating@mit.edu

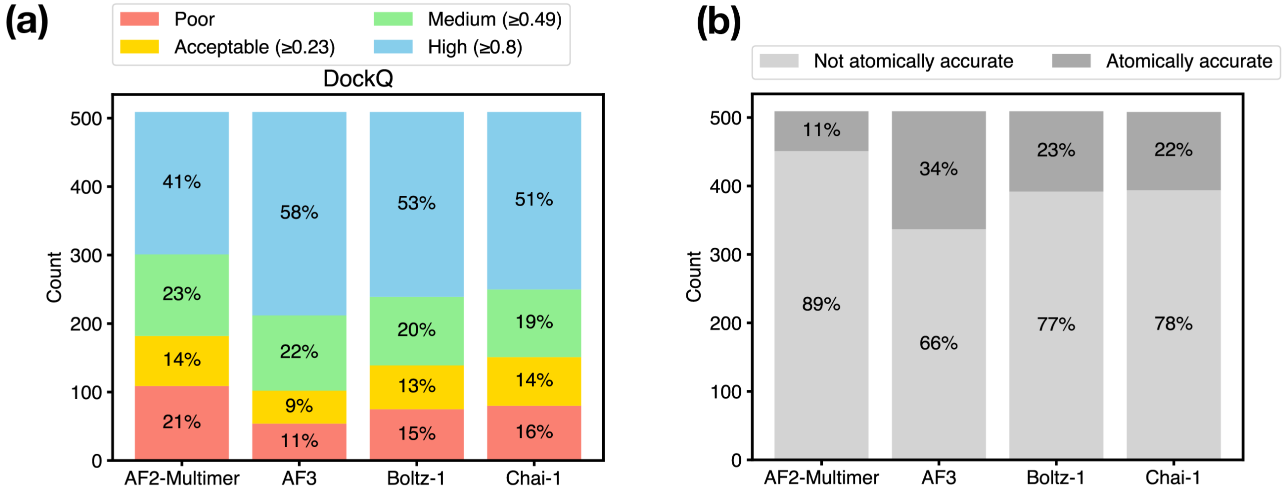

Figure S1. Summary of docking accuracy. **(a)** Fraction of predictions in each DockQ bin, using categories from the CAPRI assessment [1, 2, 3]. **(b)** Fraction of atomically accurate predictions, as defined by the following criteria: >90% of native contacts recovered (contacts within 4 Å), no clashes, peptide all-atom RMSD < 2 Å, interface all-atom RMSD < 3 Å.

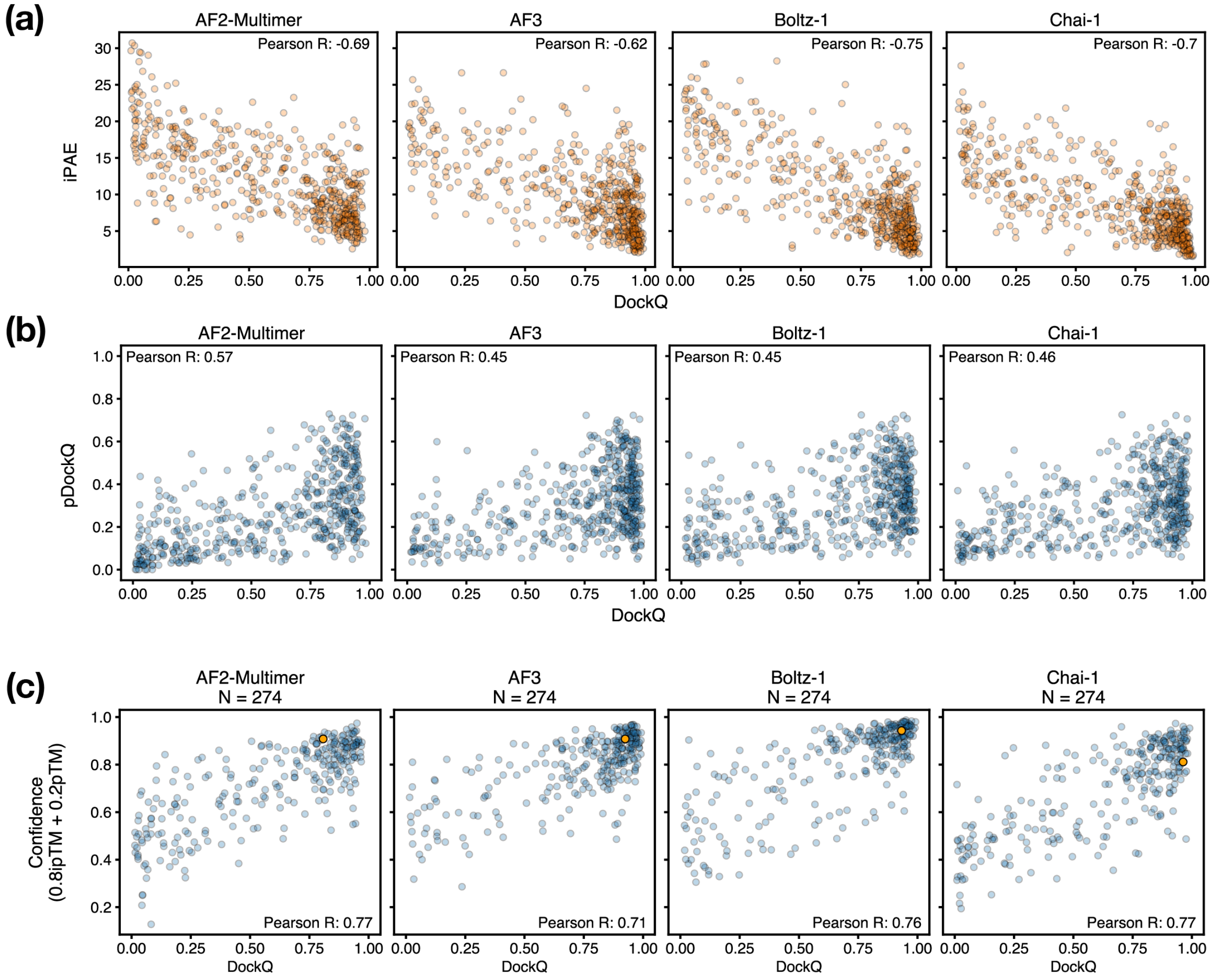

Figure S2. **(a)** Model confidence and accuracy as measured by interaction PAE (iPAE) and DockQ. **(b)** Model confidence and accuracy as measured by pDockQ and DockQ. **(c)** Model confidence and DockQ for N=274 dataset (resulting from clustering the dataset at target protein TM-score ≥ 0.5). The representative Retinoid X receptor (CATH superfamily 1.10.565.10) complex is shown in orange.

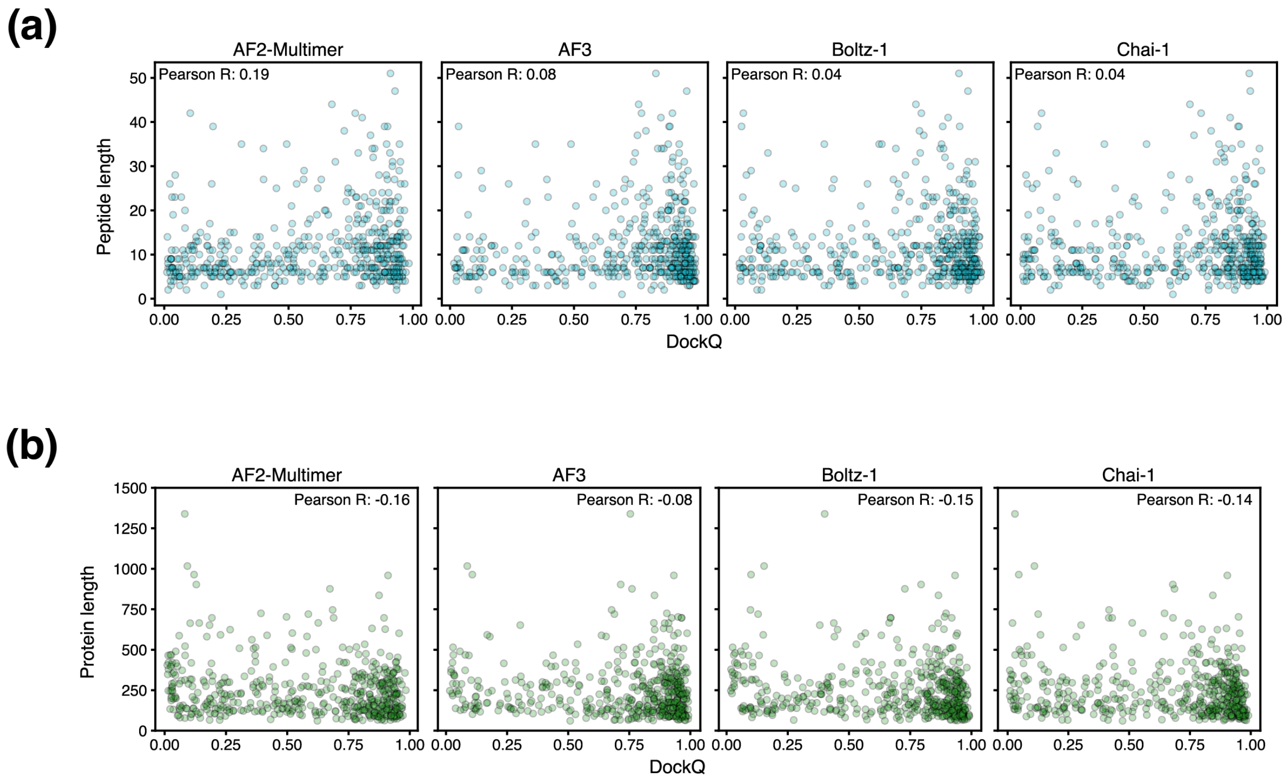

Figure S3. Chain lengths and prediction accuracy as measured by DockQ.

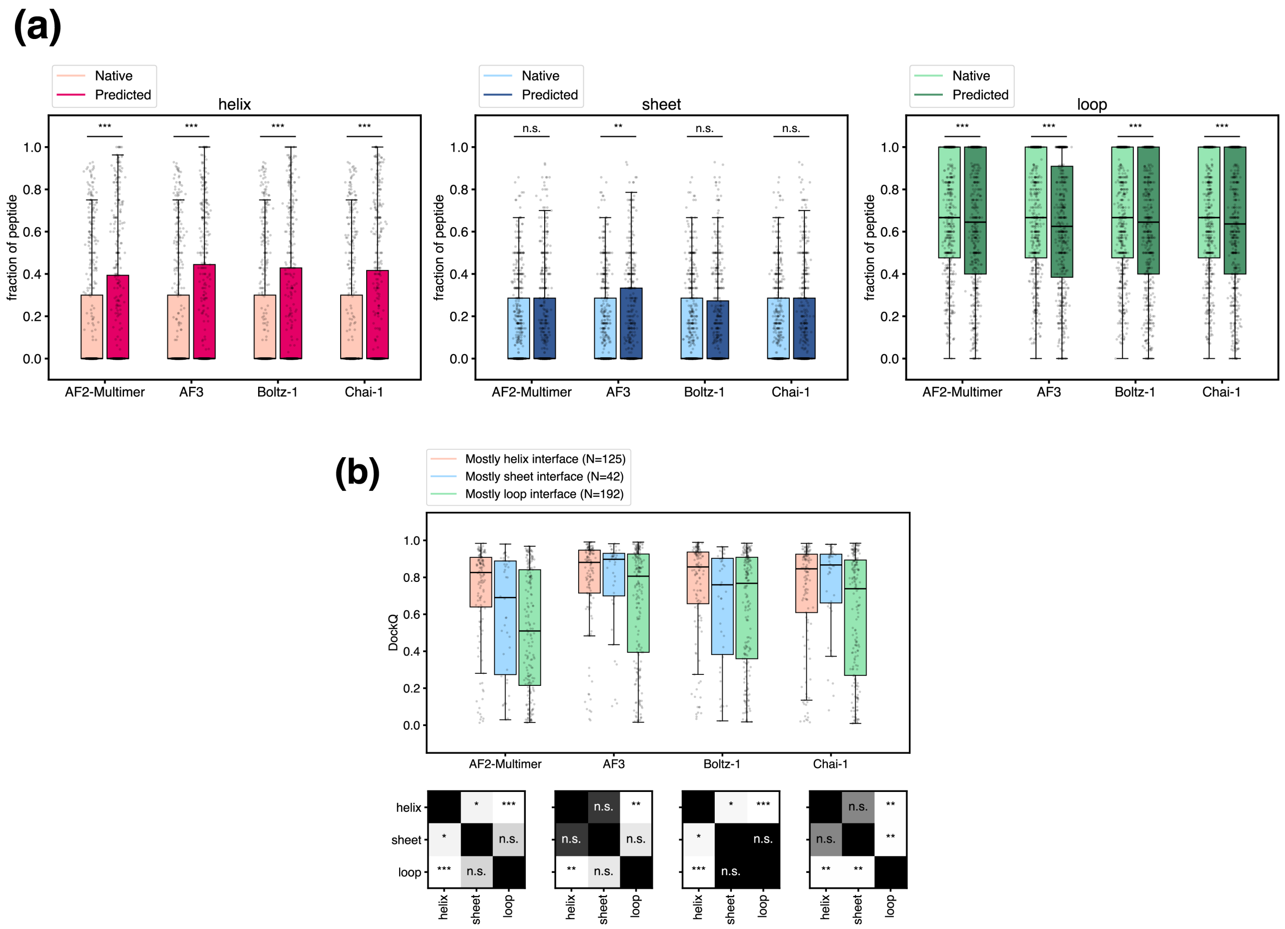

Figure S4. Secondary structure biases in predicted complex structures. **(a)** Secondary structure composition of native peptides and peptide structures predicted by the different models. The fraction of peptide residues adopting a certain secondary structure is shown. (Wilcoxon signed-rank test, *$p < 0.05$, **$p < 0.01$, ***$p < 0.001$). **(b)** Distribution of DockQ scores for complexes where the native interface had >50% residues with helical, sheet, or loop-like secondary structure. *p*-values shown in table: Mann-Whitney U-test, *$p < 0.05$, **$p < 0.01$, ***$p < 0.001$.

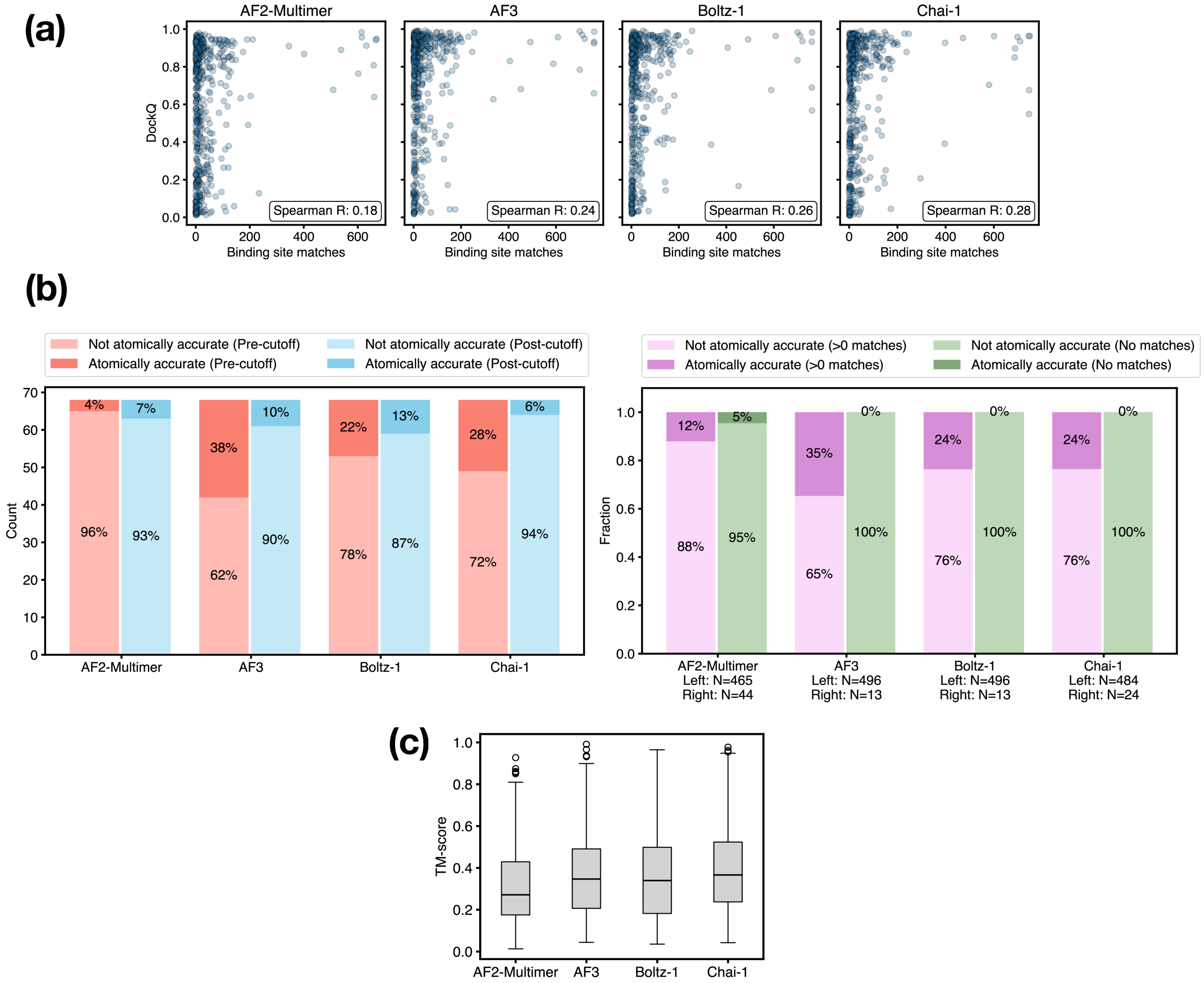

Figure S5. Prediction accuracy is higher for structures with similarity to the training set. **(a)** DockQ scores vs. the number of binding site matches. **(b)** Fraction of atomically accurate predictions for the (left) pre-cutoff and post-cutoff sets, N = 68 for each, and (right) the examples with > 0 binding-site matches in the training set vs. 0 training-set matches. Atomic accuracy is defined by the following criteria: >90% of native contacts recovered (contacts within 4 Å), no clashes, peptide all-atom RMSD < 2 Å, interface all-atom RMSD < 3 Å. **(c)** Distribution of TM-score values between the peptide predicted alone and the native bound peptide structure.

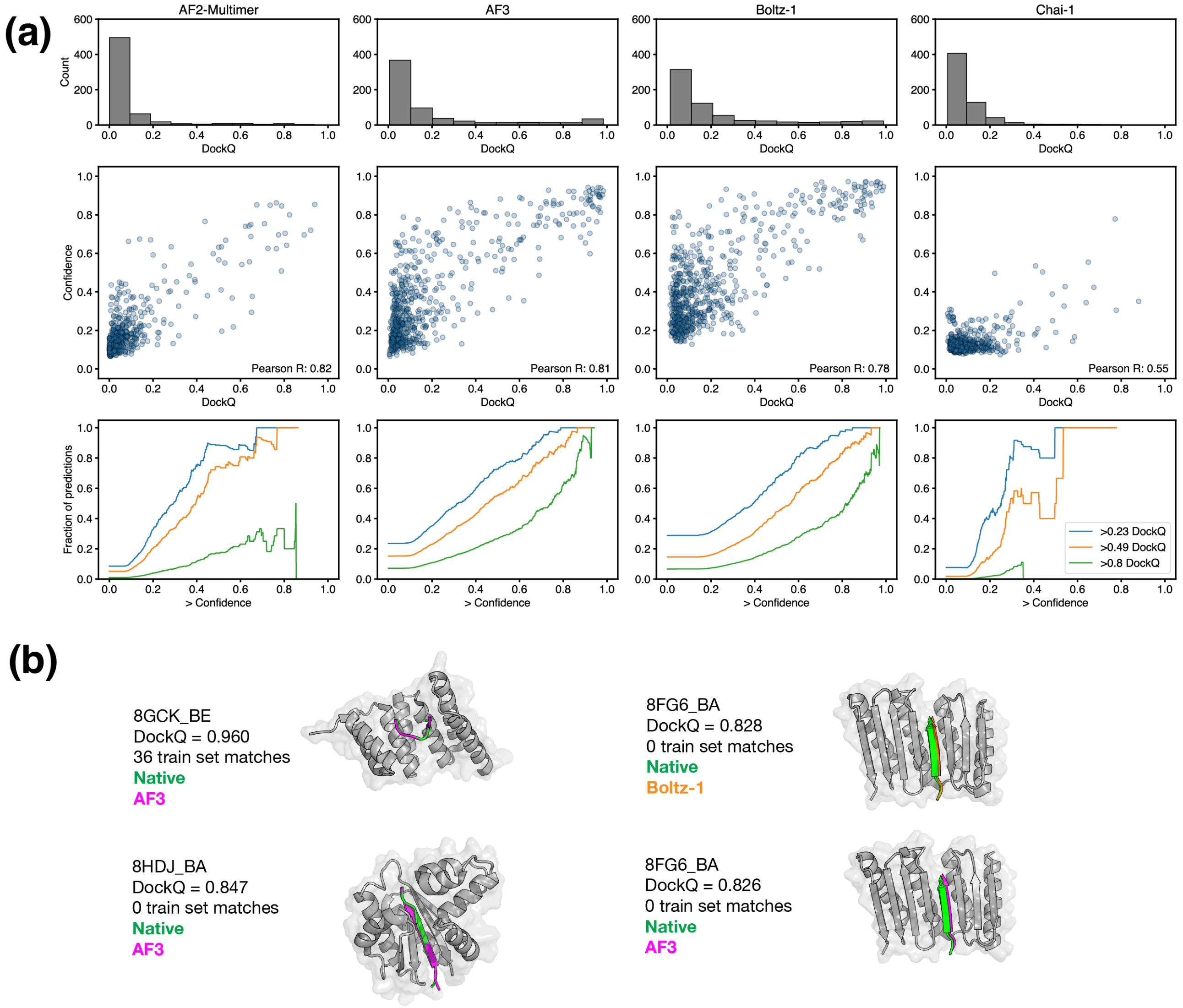

Figure S6. Predictions are rarely accurate without an MSA. **(a)** Model confidence and accuracy when no MSA was provided for either chain. **(b)** Complexes where MSA-ablated predictions had DockQ > 0.8 and the structures were released after each model's respective training set date cutoff. Binding-site matches in the training sets were determined through the same procedure used in Figure 2c (Methods, "Search for interaction matches in the PDB").

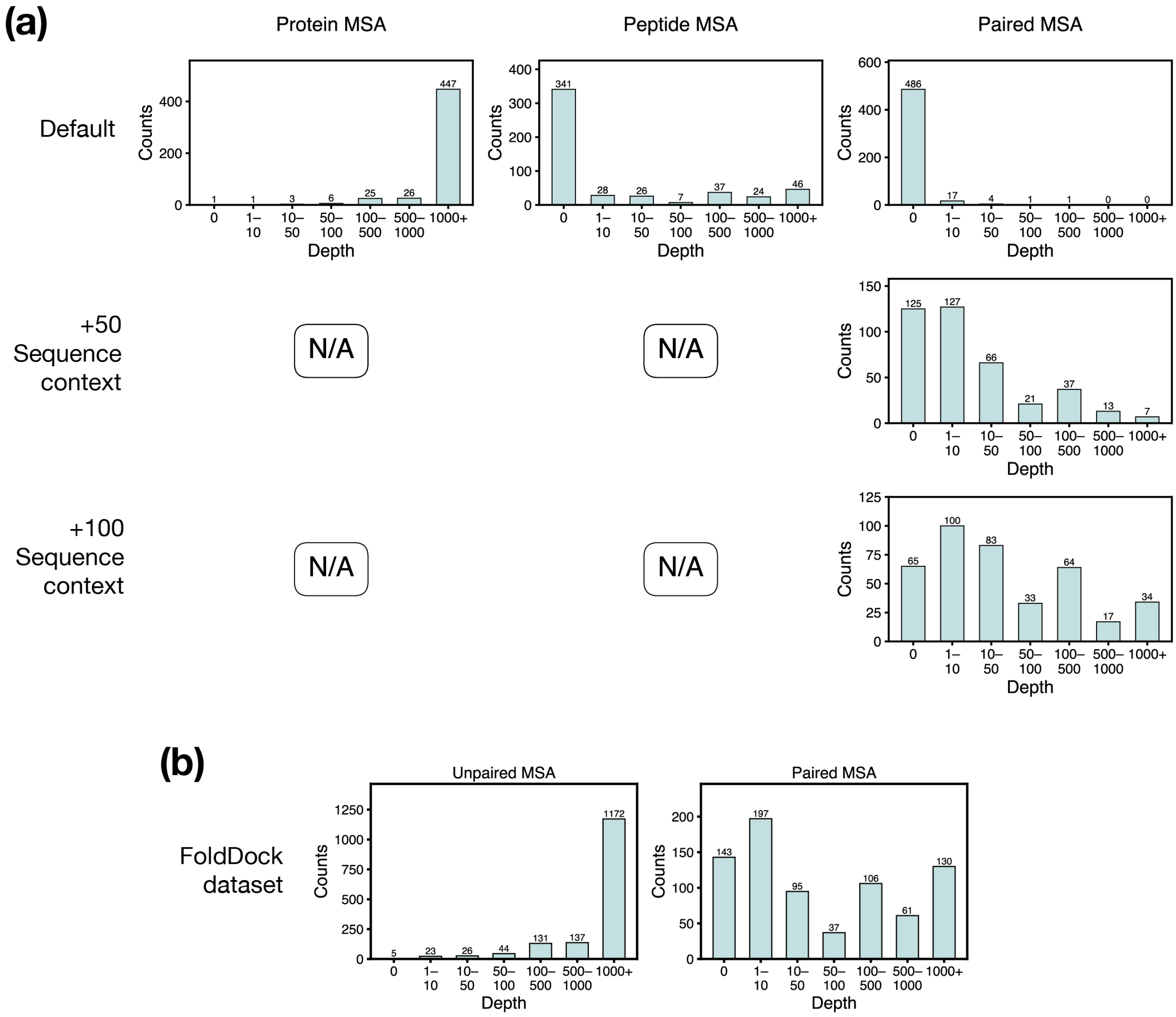

Figure S7. MSA depths for various inputs. **(a)** For "Default" predictions, protein MSA depths and peptide MSA depths correspond to MSAs generated using the mmseqs server when only an unpaired MSA was requested. The paired MSA depths correspond to MSAs generated when only a paired MSA was requested. For +50 and +100 sequence context, depths are shown only for MSAs where the peptide could be mapped to a UniProt protein; predictions were made only for examples with MSAs containing at least 50 peptide sequences that passed a quality filter (Methods, "Mapping peptide sequences to the full-length protein"). **(b)** MSA depths for the Bryant et al. PPI dataset [4]. The unpaired MSA depth is reported per-chain, while each paired MSA depth corresponds to the paired MSA of a complex. All MSA depths are shown, though predictions were made on only those containing at least 50 sequences.

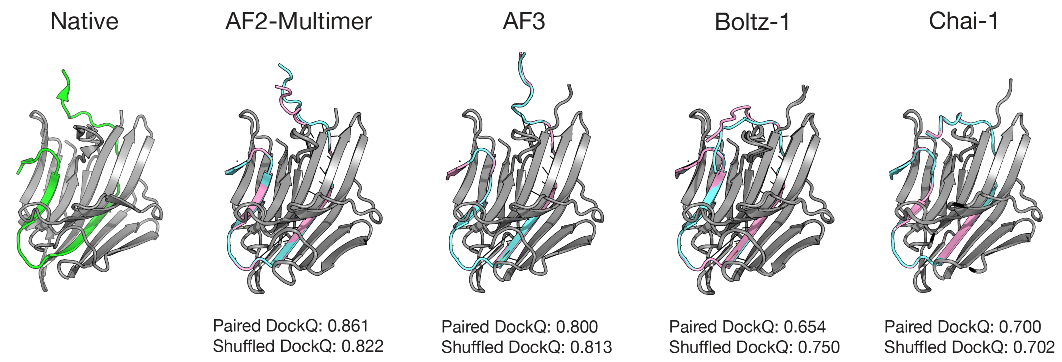

Figure S8. Predictions for 3TRS_BA with +100 context paired MSA (cyan) and +100 context shuffled MSA (pink)

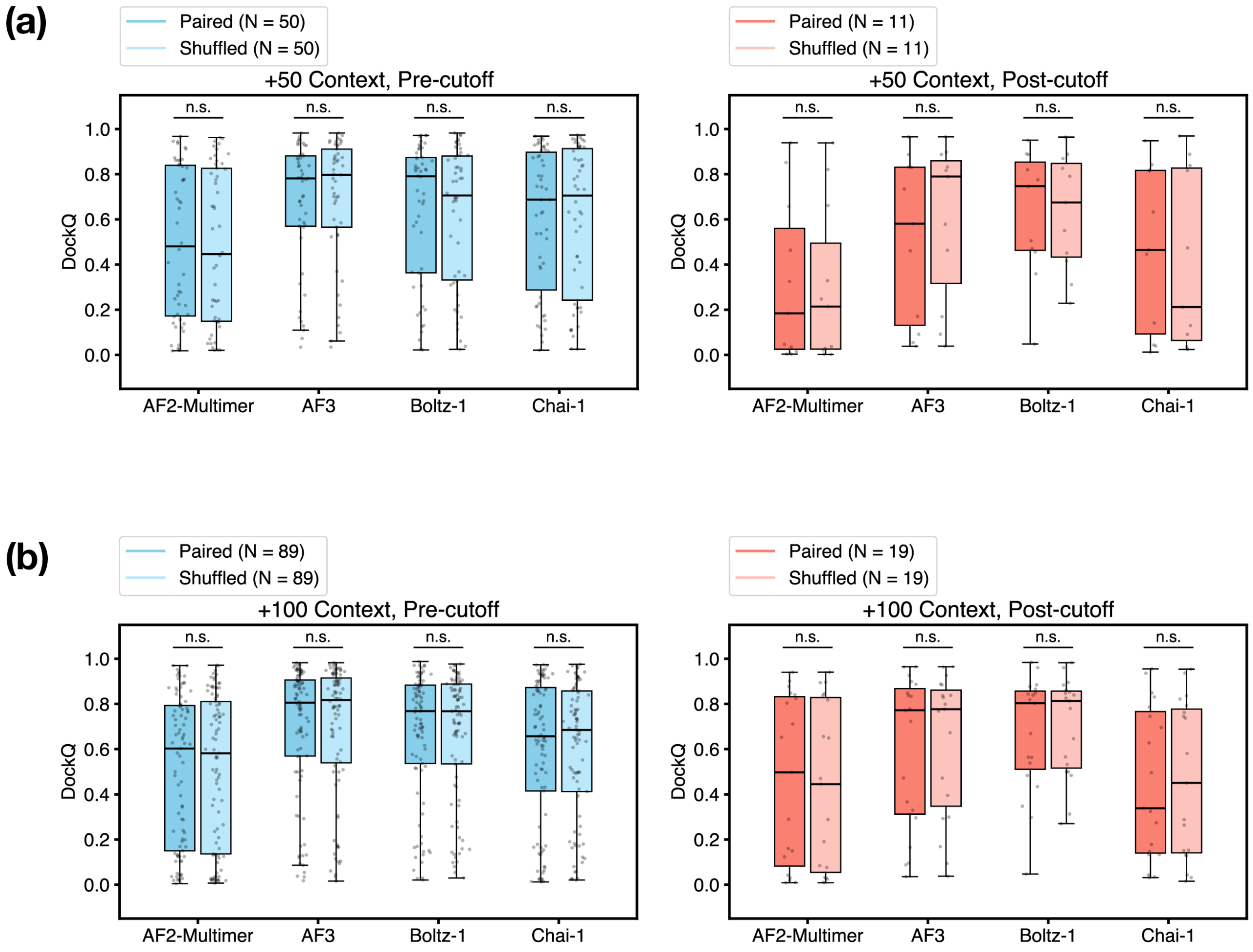

Figure S9. Distribution of MI and DockQ scores for predictions made with **(a)** +50 context and **(b)** +100 context paired vs. shuffled MSAs in the pre- and post-training date cutoff sets as described in Figure 2a (Wilcoxon signed-rank test, *$p < 0.05$, **$p < 0.01$, ***$p < 0.001$).

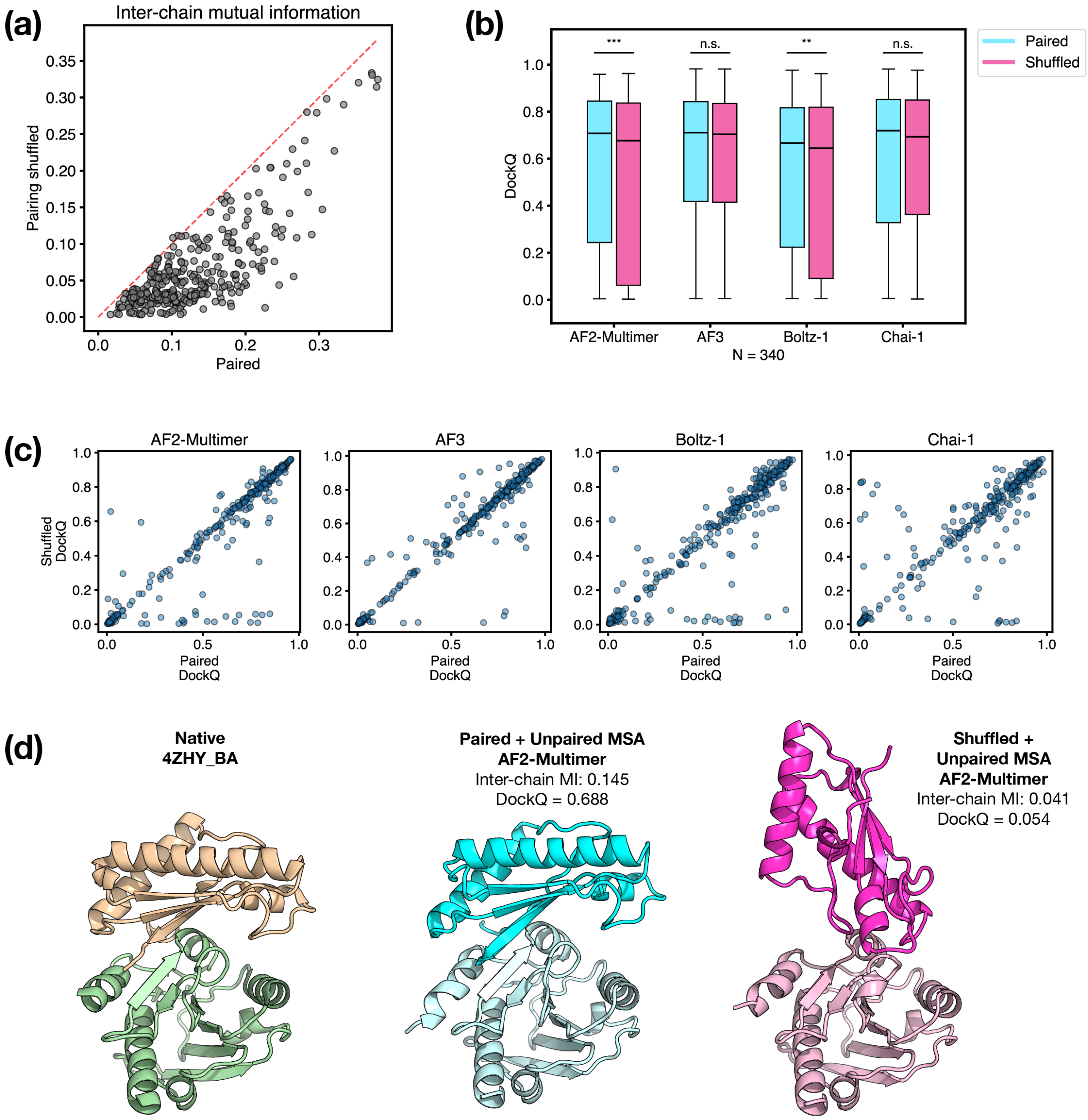

Figure S10. MSA pairing does not improve prediction performance for protein-protein interactions. **(a)** Distribution of MI for predictions made with an unpaired + paired MSA and unpaired + shuffled MSA for protein-protein complexes reported in Bryant et al. [4]. Complexes where the paired MSA had a depth of at least 50 sequences were included. (b) Distribution of DockQ scores for predictions made with unpaired + paired vs. unpaired + shuffled MSAs (Wilcoxon signed-rank test, $^{*}p < 0.05$, $^{**}p < 0.01$, $^{***}p < 0.001$). **(c)** DockQ scores for predictions made with unpaired + paired vs. unpaired + shuffled MSAs. **(d)** The native structure and AF2-Multimer predictions for 4ZHY_BA with paired + unpaired MSA and shuffled + unpaired MSA [5, 6].

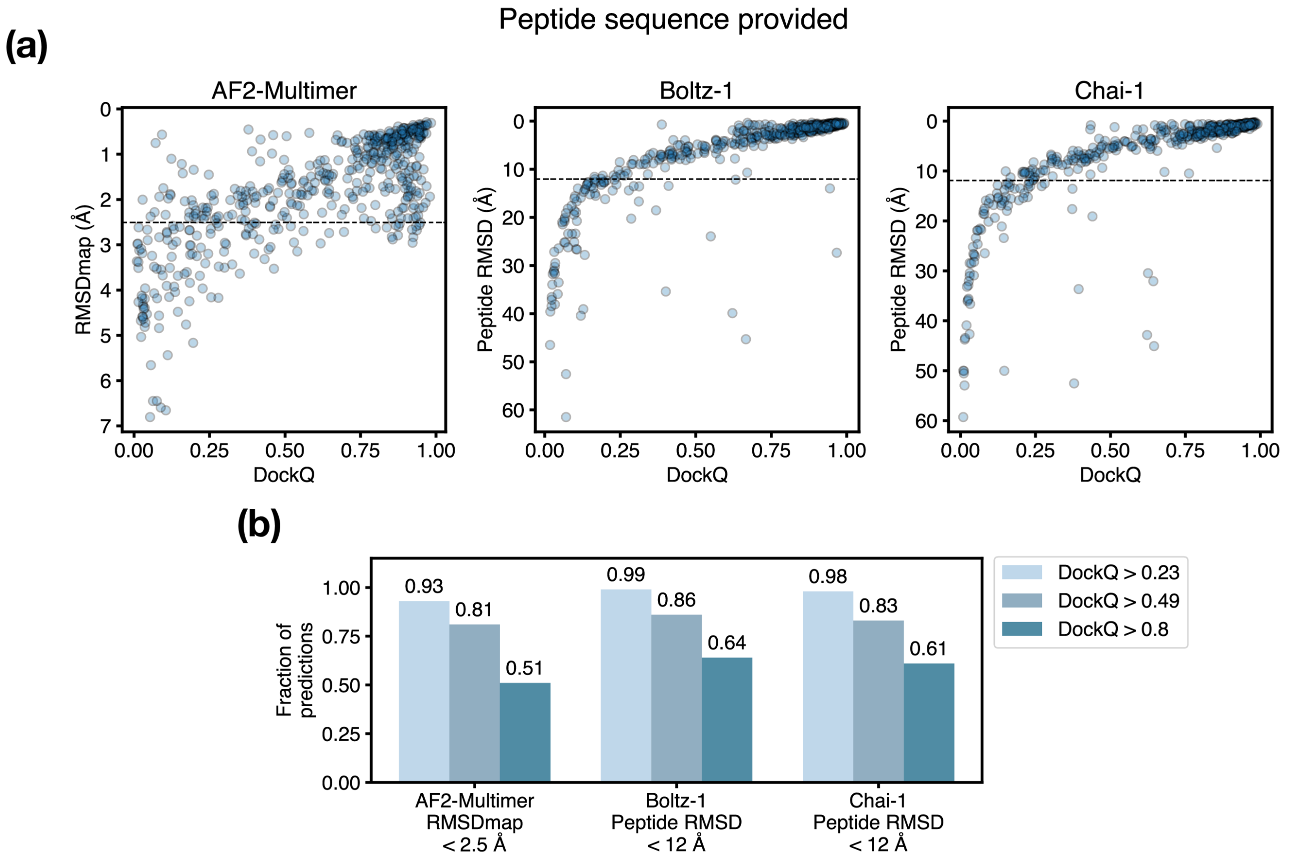

Figure S11. $RMSD_{map}$ and peptide RMSD correlate with DockQ. **(a)** Performance metric vs. DockQ for predictions where the peptide sequence was provided. For AF2-Multimer, the performance metric is contact map RMSD ($RMSD_{map}$). For Boltz-1 and Chai-1, the performance metric is peptide RMSD. **(b)** Fraction of predictions with DockQ > z, for different z, using an $RMSD_{map}$ cutoff of 2.5 Å and with a peptide RMSD cutoff of 12 Å.

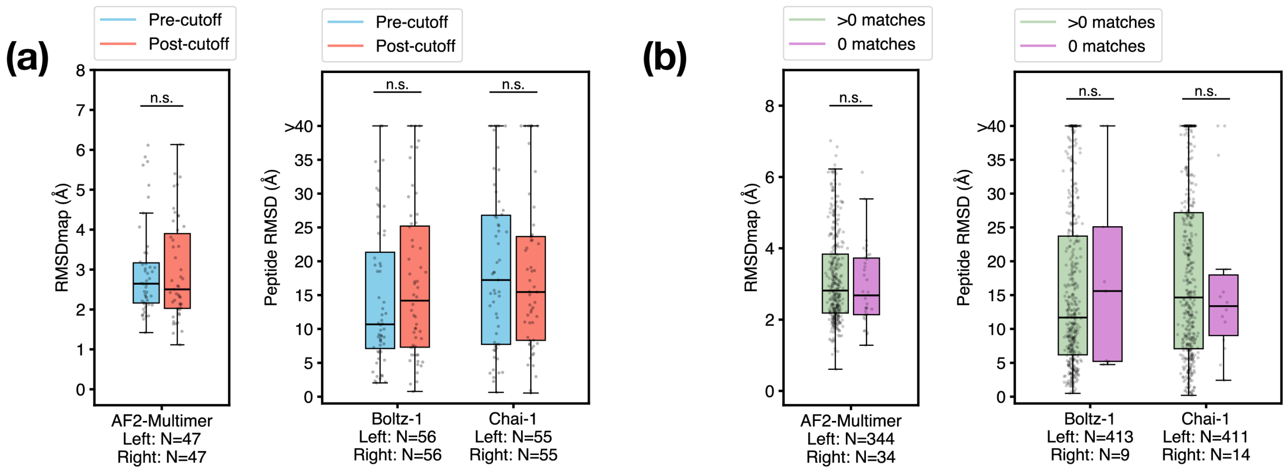

Figure S12. $RMSD_{map}$ and peptide RMSD for masked peptide predictions according to similarity to the training set. **(a)** Masked prediction $RMSD_{map}$ and peptide RMSD values for pre-cutoff and post-cutoff test sets. **(b)** Masked prediction $RMSD_{map}$ and peptide RMSD values for complexes with >0 binding-site matches or 0 binding-site matches. For both panels, only complexes for which unmasked predictions were successful, according to the designated metric, are shown.

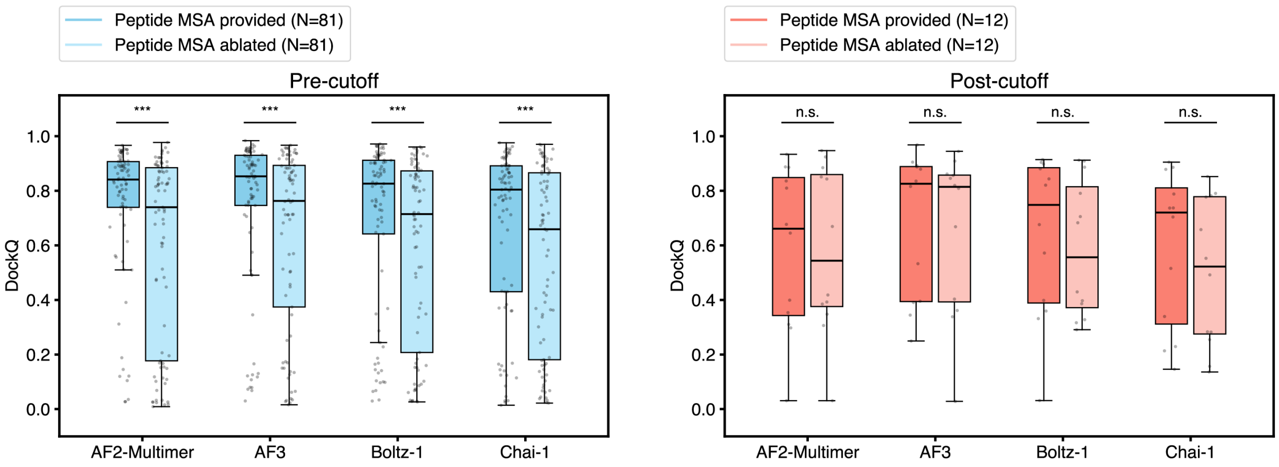

Figure S13. Influence of the peptide MSA on prediction accuracy. Comparison of DockQ between using the unpaired MSA vs. the peptide-ablated MSA for complexes where the peptide MSA had at least 50 sequences in the pre- and post-training date cutoff set as described in Figure 2a (Wilcoxon signed-rank test, *$p < 0.05$, **$p < 0.01$, ***$p < 0.001$).

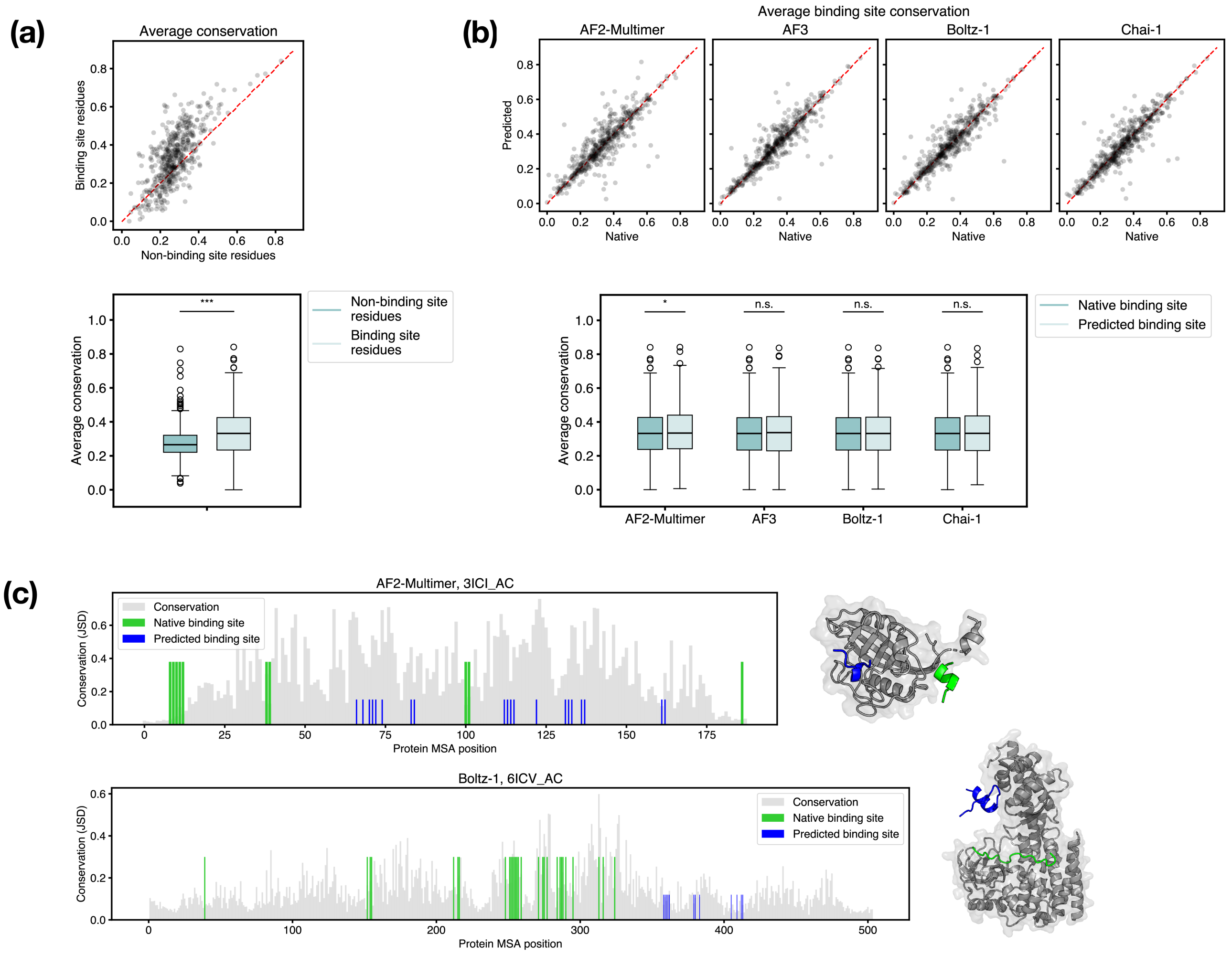

Figure S14. Predicted binding sites are not more conserved than native binding sites. **(a)** Average binding site conservation of native binding site residues vs. native non-binding residues. (Mann-Whitney U-test, *$p < 0.05$, **$p < 0.01$, ***$p < 0.001$). **(b)** Binding site conservation of predicted and native structures. All p-values are from a Wilcoxon signed-rank test comparing the two distributions. **(c)** Examples of conservation of binding-site residues on the protein for native and predicted structures.

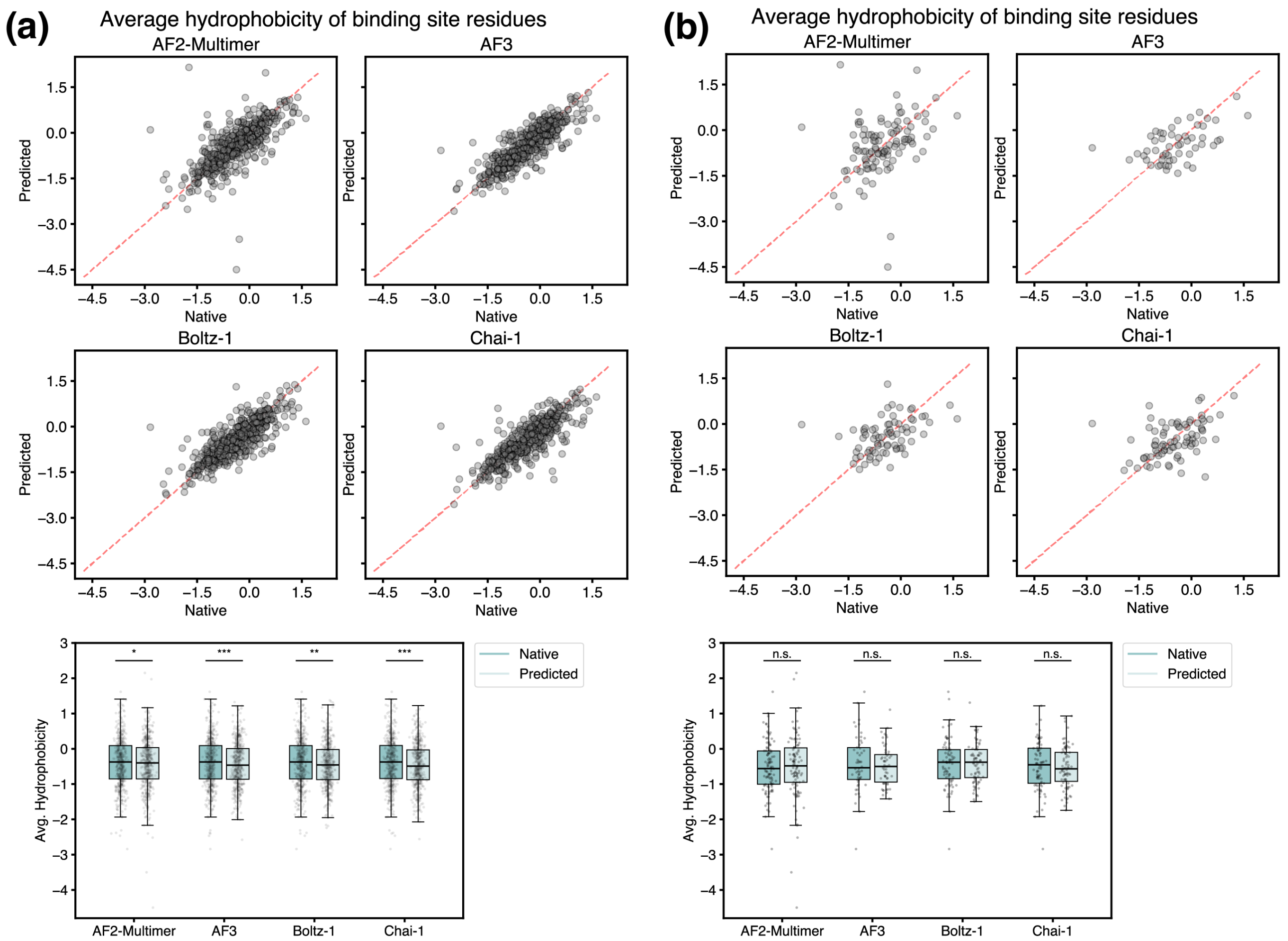

Figure S15. The predicted binding site is not determined by hydrophobicity. **(a)** Average hydrophobicity of binding-site residues in native and predicted structures. **(b)** Average hydrophobicity of binding-site residues in native and predicted structures for predictions with DockQ < 0.23. (Wilcoxon signed-rank test, *$p < 0.05$, **$p < 0.01$, ***$p < 0.001$).

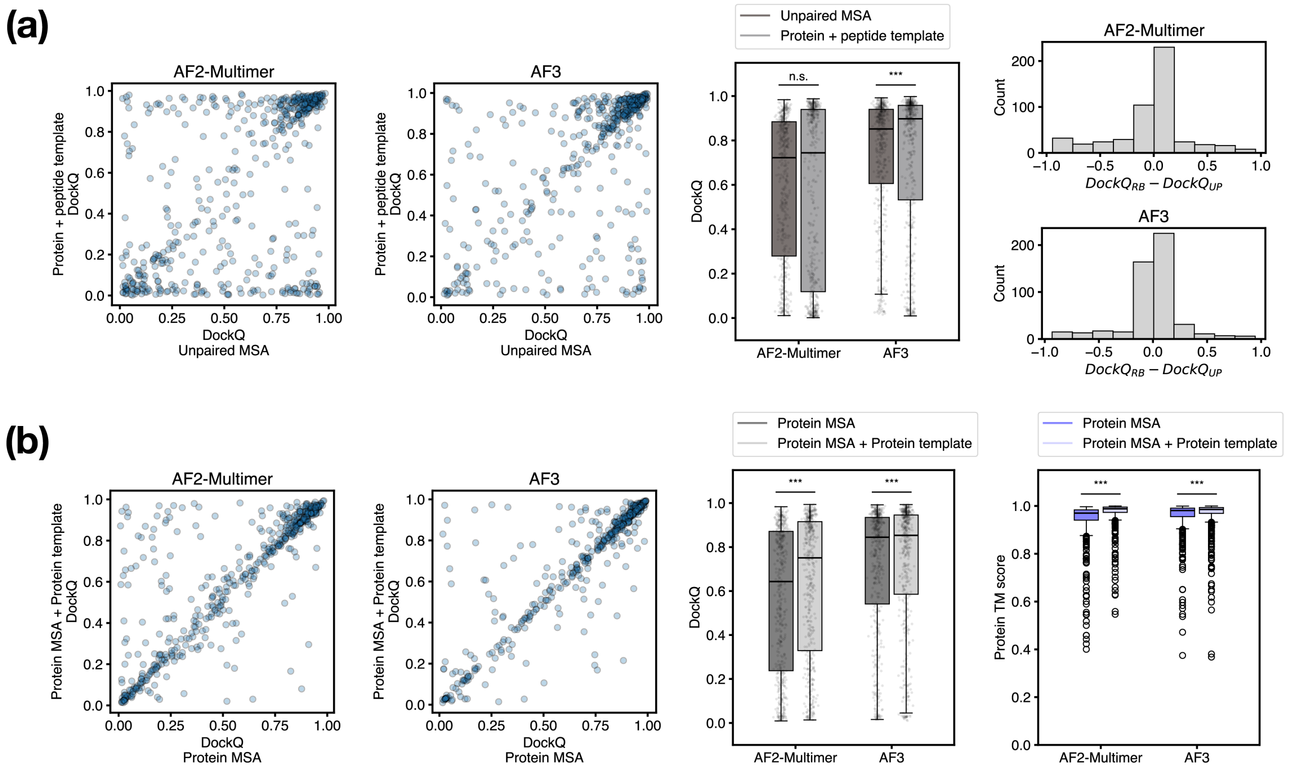

Figure S16. Impact of using a structural template as input to the predictions. **(a)** DockQ scores for predictions made with an unpaired MSA or with templates provided for each chain and no MSAs. The box plots and histograms summarize the differences in DockQ scores when using template inputs vs. unpaired MSA input for AF2-Multimer and AF3. **(b)** DockQ scores for predictions made with just the protein MSA or with the protein MSA + template; the peptide was provided as a sequence with no template or MSA. The box plots and histograms summarize the differences in DockQ scores and protein TM-scores for the two conditions. (Wilcoxon signed-rank test, *$p < 0.05$, **$p < 0.01$, ***$p < 0.001$).

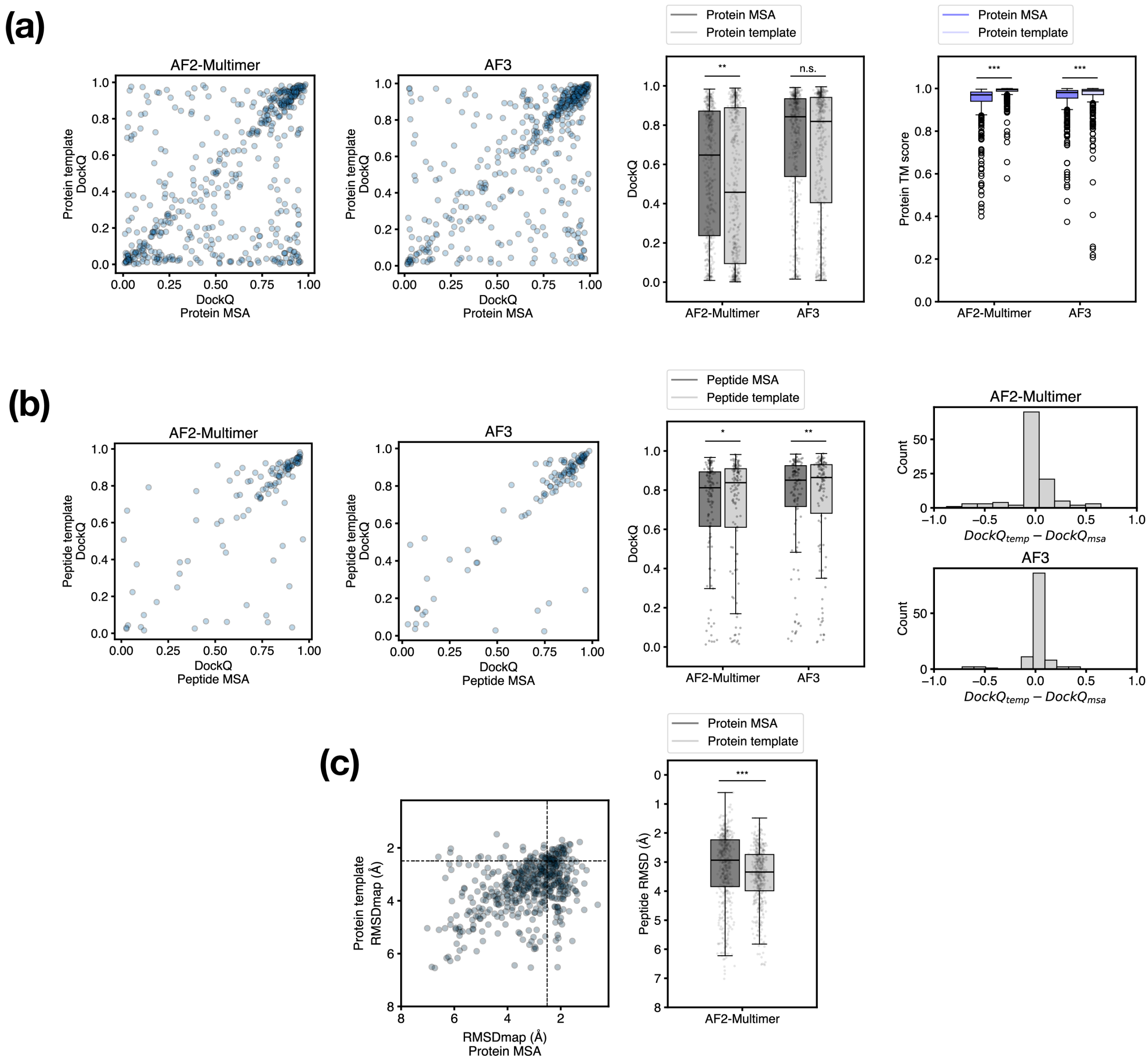

Figure S17. The influence of input templates vs. MSAs on prediction performance. **(a)** DockQ for predictions made with just the protein MSA or with just the protein template as input; the peptide sequence was provided without template or MSA. The box plots summarize the differences in DockQ scores and protein TM-scores when using just protein template vs. just the protein MSA as input for AF2-Multimer and AF3. **(b)** DockQ for predictions made using the unpaired MSA (protein MSA + peptide MSA) or with the protein MSA + peptide template. The box plots and histograms summarize the differences in DockQ scores when using the unpaired MSA vs. protein MSA + peptide template for AF2-Multimer and AF3. (Wilcoxon signed-rank test, *$p < 0.05$, **$p < 0.01$, ***$p < 0.001$). **(c)** $RMSD_{map}$ values for masked predictions (peptide sequence consisting of tokens "X") made with just the protein MSA or with just the protein template as input.

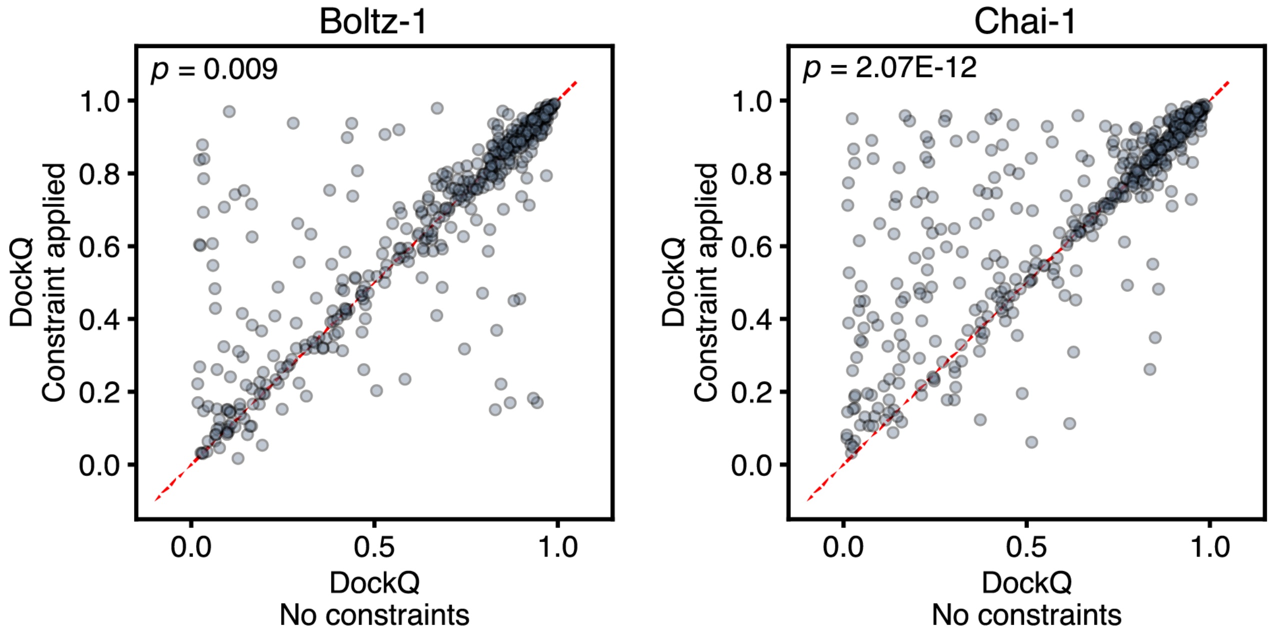

Figure S18. Model accuracy when pocket input for Boltz-1 or restraint input for Chai-1 are provided. *p*-values reflect the difference in DockQ distributions between constrained prediction and unconstrained prediction (Wilcoxon signed-rank test).

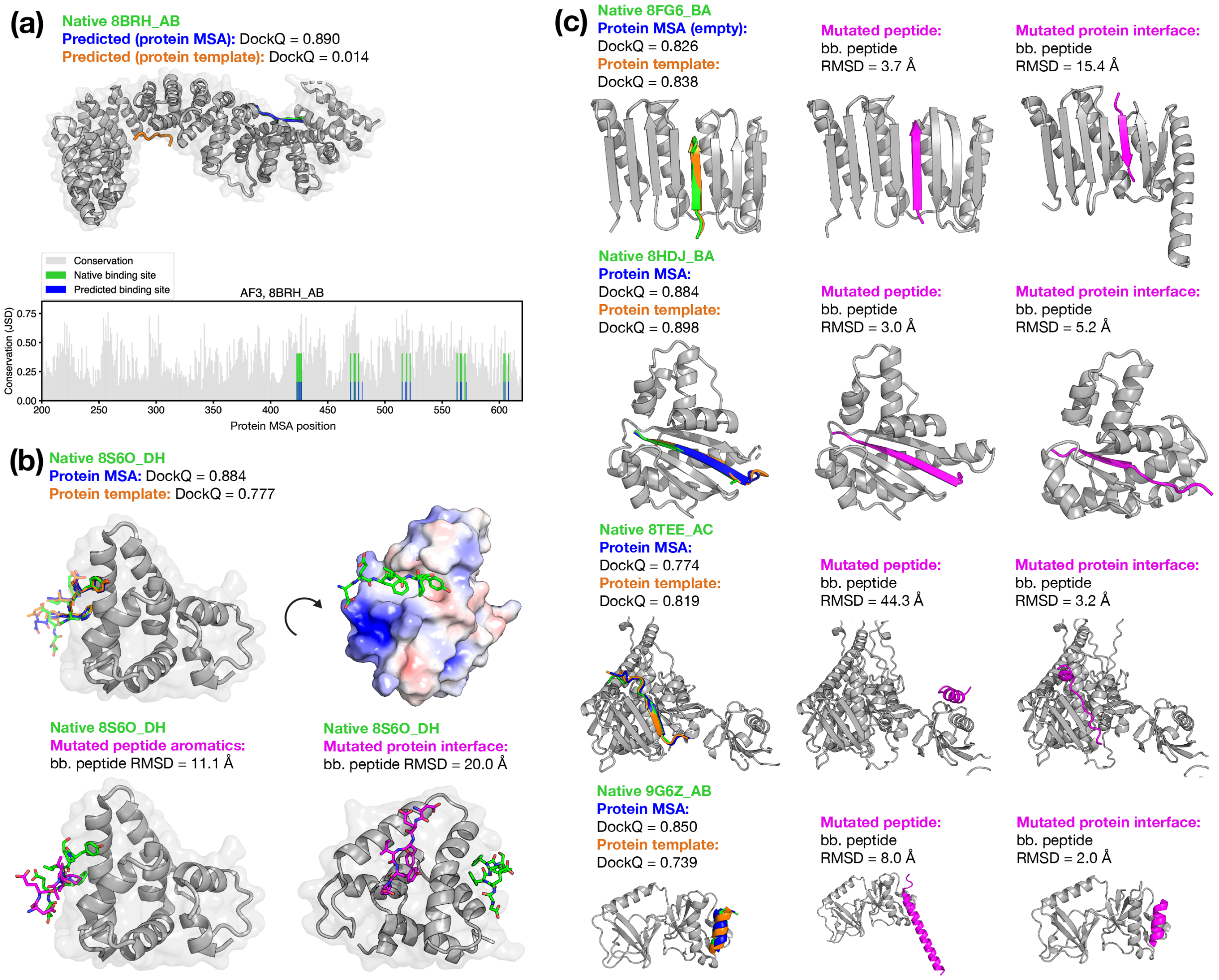

Figure S19. Details for successfully predicted complexes lacking binding-site matches in the training sets. **(a)** Top: AF3 predictions for 8BRH_AB made with the protein MSA input vs. protein template as input; no peptide MSA. Only the native protein structure is shown for clarity. Bottom: Positional conservation of protein residues with native and predicted binding sites indicated. Only 8BRH_A positions 200-620 are shown for clarity. The prediction shown was made using the protein MSA with no template. **(b)** Top left: AF3 predictions for 8S6O_DH made with only the protein MSA input vs. only the protein template input; top right: A rotated view of the native structure with APBS electrostatics shown on the protein surface; bottom left: AF3 prediction for 8S6O_DH where aromatics in the peptide were mutated to alanine; bottom right: A rotated view of the AF3 prediction for 8S6O_DH where residues corresponding to the native binding site were mutated to alanine. **(c)** AF3 predictions for 8FG6_BA, 8HDJ_BA, 8TEE_AC, and 9G6Z_AB where "mutated peptide" corresponds to a peptide where interface residues were mutated to alanine and "mutated binding site interface" corresponds to a protein where interface residues were mutated to alanine. For 9G6Z_AB, only the region corresponding to the resolved peptide residues in the native PDB structures is shown, except for the mutated peptide prediction, where the peptide corresponding to the whole input peptide sequence is shown.

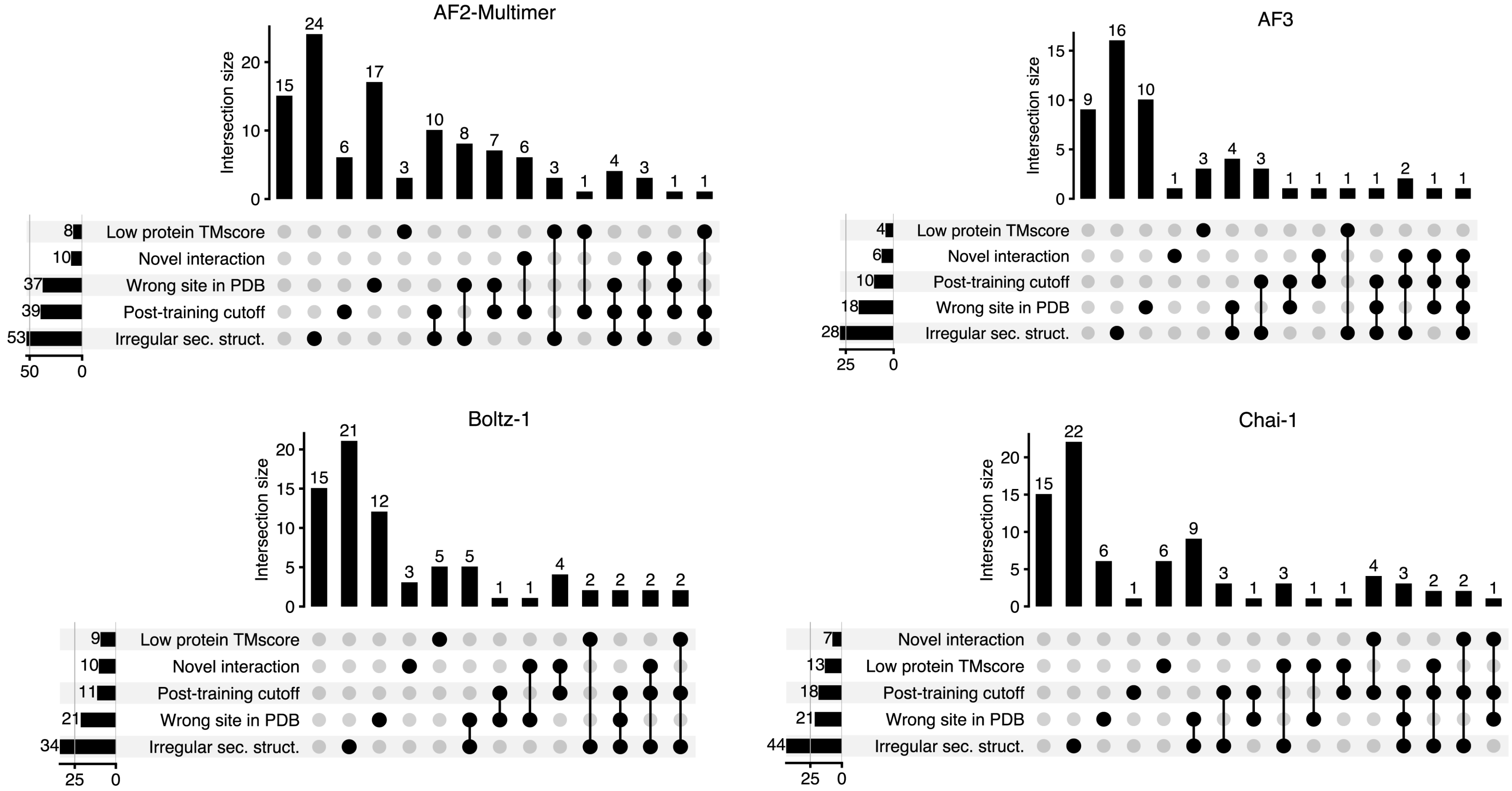

Figure S20. Upset plots for all poor predictions (DockQ < 0.23) using default model settings (unpaired MSA, no templates). A TM-score ≤ 0.6 is considered poor. A novel interaction is one with 0 binding-site matches in the training set. Training date cutoffs for this analysis were defined separately for each model. An incorrect prediction for which there is at least one match in the training set is labeled as "Wrong site in PDB." Irregular secondary structure is defined by native interface residues having >50% loop-like residues.

Supplementary Tables

Table S1. Comparison of settings and success rates between different studies that benchmarked structure prediction models on protein-peptide complexes.

	AF Model	Dataset size	Glycine linker	Separate chains	Delimited peptide	Success definition	Success rate
Tsaban et al. (2022)	Monomer v2.0	96 (Large non-redundant set)	Yes	No	Yes	Interface RMSD < 2.5 Å	38.3%
Tsaban et al. (2022)	Monomer v2.0	96 (Large non-redundant set)	No	Yes	Yes	Interface RMSD < 2.5 Å	51%
Ko and Lee (2021)	Monomer v2.0	203 (PepBDB)	Yes	No	Yes	Peptide RMSD < 2.0 Å	51% (best of 5)
Ko and Lee (2021)	Monomer v2.0	203 (PepBDB)	Yes	No	Yes	Peptide RMSD < 2.0 Å	44% (highest confidence)
Bret et al. (2024)	Multimer v.2.2	42	No	Yes	No	DockQ > 0.23	40%
Bret et al. (2024)	Multimer v.2.2	42	No	Yes	Yes	DockQ > 0.23	76%
Johansson-Åkhe and Wallner (2022)	Multimer v.2.2	112	No	Yes	Yes	DockQ > 0.23	59%
Lee et al. (2024)	Multimer v2.2	136	No	Yes	Yes	DockQ > 0.23	88%
Zhou et al. (2025)	Multimer v2	Unknown	No	Yes	Yes	Fnat $\geq$ 0.8	53%
Zhou et al. (2025)	AlphaFold3	Unknown	No	Yes	Yes	Fnat $\geq$ 0.8	76.8%
Zhou et al. (2025)	Protenix	Unknown	No	Yes	Yes	Fnat $\geq$ 0.8	80.8%
Zhou et al. (2025)	Boltz-1	Unknown	No	Yes	Yes	Fnat $\geq$ 0.8	71.7%
Zhou et al. (2025)	Chai-1	Unknown	No	Yes	Yes	Fnat $\geq$ 0.8	78.8%

Methods

Dataset

We queried the PDB on December 12th, 2024, for structures composed of $\geq$ 2 chains in the biological assembly with resolution below 2.5 Å and no DNA or RNA. Further filtering was done using a combination of Biopython and PyMol Python API, with filtering steps largely inspired by the curation protocol used to create the Propedia database [13, 14, 15, 16]:

1. The peptide chain must have $\geq$ 5 and $\leq$ 50 residues and the protein chain must have $\geq$ 60 residues.
2. At least one peptide atom must be within a distance of 6Å from any receptor atom.
3. The protein–peptide complex must have an interface area of >100 Å^2. Interface area was calculated as IA $= ASA(\text{prot}) + ASA(\text{pep}) - ASA(\text{complex})$ [17]. $ASA(\text{prot})$ and $ASA(\text{pep})$ are the accessible surface area values for the protein and peptide chains alone, respectively. $ASA(\text{complex})$ is the accessible surface area of the complex.
4. There are no cofactors or ligands within 6 Å of any peptide atom. Common solvents and salts were not considered cofactors or ligands.
5. The peptide has one or fewer non-canonical or post-translationally modified amino acids.
6. For filtering out interactions mediated by a third protein:
   (a) There is no other chain making a >100 Å^2 interface with the peptide.
   (b) There is no other chain making a >500 Å^2 interface with the protein.
7. There are no crystal contacts between the peptide and any other protein chain with area greater than the interaction interface between the target protein and peptide.

Complexes were then clustered to remove highly similar interactions. First, the protein chains were clustered with foldseek [18] to identify groups of complexes with a highly similar protein. Cluster members shared TM-score $\geq$ 0.9 with 90% of residues aligned. Then, within each cluster, the pairwise RMSD between all peptides was calculated (after structures were aligned to the protein). Hierarchical clustering based on the pairwise RMSDs, using scipy linkage and fcluster, was used to find subgroups of similar interactions within protein clusters, generating "peptide clusters." Linkage flattening (i.e., forming flat clusters from the hierarchical clustering) was done at threshold = 10 Å, such that the maximum RMSD allowed between any two clusters before merging is 10 Å. One representative structure from each of 509 peptide clusters was included in the final dataset. A table describing the dataset is included in the data deposition and reports the PDB code and relevant chains for each complex, the number of inter-chain contacts, any ligands present in the PDB structure, interface area between the protein and peptide, the interface area between the peptide and any crystallization contacts, protein cluster information, CATH superfamily annotations, and peptide cluster information.

We also tried clustering at a much lower protein-similarity threshold (TM-score $\geq$ 0.5) and selected one representative from each cluster. This resulted in a less redundant dataset at the protein family level of just 274 complexes. However, we found that this dataset obscures the range of accuracies and confidence scores within the clusters of our chosen test set (with size 509), supporting that the examples that we included are not redundant despite sharing a similar target protein fold. For example, we looked at all complexes where the protein was a Retinoid X receptor (CATH superfamily 1.10.565.10). In the N=509 dataset, we see a range of accuracies, from DockQ score 0.50 to 0.98. When only one representative is chosen, this variability is lost (S2c). Considering the tradeoffs between representation and family redundancy, we chose the test set with 509 complexes.

Base predictions

For benchmarking, we used the LocalColabFold [19, 20] implementation of AF2-Multimer v2.2, AlphaFold3 v3.0.1, Boltz-1 commit d99ceaa61f3af29a4feabee063e5bba8b3f95eb8, and Chai-1 commit 71eff6ac945db726e1d18f4c9e70fba85cd49688. AF2-Multimer v2.2 was used instead of v2.3 to enable comparison to other studies benchmarking AF2-Multimer on protein-peptide complexes. Chai-1 was used without ESM embeddings to enable evaluation of all models with and without an MSA. An MSA for each chain was constructed using colabfold, which made API calls to the mmseqs2 server. These MSAs were reused for prediction with Boltz-1 and Chai-1. All models were run with 3 recycling steps, and Boltz-1 and Chai-1 were run with 200 diffusion timesteps (the default setting). AlphaFold3 was run with default settings. DockQ was calculated using DockQ v2.8.

All-atom peptide RMS and interface RMS values were calculated with a modified version of the DockQ v2 repo that calculates all-atom DockQ: https://github.com/lindseyguan/DockQ_allatom. Interaction PAE was calculated using the method described for the model AF2 Initial-Guess [21], which averages protein-aligned PAE and peptide-aligned PAE. DSSP v3.1.4 was used to calculate secondary structure [22, 23].

Inter-chain attention masking

During the attention mechanism, an attention matrix is calculated that captures how much each position in the input should attend to every other position. The attention matrix has shape $N \times N$, where N is the total length of the protein-peptide complex. For the results in Figure 6, a mask of $-\infty$ was added to the positions corresponding to inter-chain attention, i.e., the off-diagonal. Inter-chain attention masking was implemented in a fork of the OpenFold repository [24]: https://github.com/lindseyguan/openfold.

Templates

Templates were created from the native complex structures. New PDB files were created for each chain, with the entire chain randomly rotated and translated using the Python PyMol API to ensure that models cannot extract the relative docked positions from template coordinates [14].

Peptide sequence-masked predictions

Masked peptide predictions were made by replacing all peptide residues with the masked token ‘X’ or ‘G,’ maintaining the length of the original peptide. The contact maps for AF2-Multimer predictions were extracted from the distograms. The RMSD_{map} score is based on pairwise distances between each residue in the protein and each residue in the peptide. Peptide RMSD for Chai-1 and Boltz-1 was calculated from the output structure using the Python PyMol API [14]. RMSD was calculated on only backbone heavy atom coordinates to enable comparison to the masked peptide residues, which lack sidechains in the model output.

Constrained predictions

For Boltz-1, the binding site was defined as protein residues in the native structure containing any atom within 4 Å of any peptide atom. Three binding-site residues were randomly selected to be pocket input. For Chai-1, one protein residue-peptide residue pair less than 4 Å apart in the native structure was randomly selected and restrained to a maximum distance of 10 Å.

Pre-training and post-training cutoff split

For post-training examples, we used all 68 structures from our test set released after the latest training cutoff date across the models, which was 2021-09-30 (for AF3 and Boltz-1). The pre-training set consisted of only the latest 68 structures from before the earliest training cutoff, which was 2018-04-30 (for AF2-Multimer).

Search for interaction matches in the PDB

For each complex, we first looked for similar instances of the target protein in the PDB (available to each method at the time of training) using structure similarity with foldseek [18] and sequence similarity with mmseqs2. Structure-based matches had a TM-score $\geq$ 0.6 with 80% of residues aligned.

Matches found for the target proteins (from either foldseek or mmseqs2) were considered "hits." We tested each hit to determine whether it participates in an interaction similar to the interaction between the target protein and peptide, using the following steps. TMalign [25] was used to structurally align the hit and query target proteins and extract a mapping between residues. Biopython was used to assess whether residues on the hit that aligned to binding site residues on the query were also binding site residues, as defined by being < 8 Å away from another protein chain. If there were at least 5 of these binding site residues ("binding site overlap"), and the corresponding protein/peptide had < 10 Å RMSD with the query peptide, then we considered the hit a binding-site match for the complex.

In summary, binding-site matches between test set complexes and PDB complexes were based on meeting three criteria:
1. Sequence- or structure-based similarity of the protein to a structure in the PDB deposited before the training date cutoff (to ensure target protein structure similarity).
2. Greater than 5 overlapping binding site residues (to ensure similar residues on the protein are participating in a binding interaction).
3. < 10 Å RMSD between the peptide and the bound protein chain in the hit (to ensure similar peptide positioning and conformation).

The foldseek and mmseqs2 queries were, respectively:

```
foldseek easy-search QUERIES_DIR pdb result tmp --exhaustive-search --alignment-type 1
-c 0.8 --cov-mode 2 --tmscore-threshold 0.6

mmseqs easy-search QUERIES.fa pdb_seqres.fa result tmp -c 0.8 --cov-mode 2
```

Python-like pseudo-code for the script used to identify binding site matches from the hits:

```
for each complex:
    query = protein structure from complex
    matches = []
    for each foldseek/mmseqs hit ('hit') for this receptor:
        check that this hit was released before the relevant training date cutoff
        query_to_hit_mapping = TMalign(query, hit)
        putative_binding_site = hit residues aligned to query binding site

        binding_site_overlap = 0
        for residue in putative_binding_site:
            nearby_atoms = BiopythonNeighborSearch(residue)
            if nearby_atoms are on another chain:
                # We consider this residue to be part of a binding site
                binding_site_overlap += 1

        binder_rmsd = backbone_RMSD(query_peptide, hit_peptide)

        # Set some cutoffs to indicate a highly similar binding site
        # We use 5 overlapping binding site residues
        # and < 10 Ang binder RMSD
        if binding_site_overlap >= 5 and binder_rmsd < 10:
            matches.append(hit)
```

Mapping peptide sequences to the full-length protein
The peptide chain from each test complex was mapped to a UniProt accession ID using the SIFTS database [26], and bulk UniProt sequences were downloaded using the ID-mapping tool: https://www.uniprot.org/id-mapping. UniProt sequences where the peptide region had > 40% mismatch to the PDB-delimited peptide were excluded. Paired MSAs were generated using the original protein sequence and the UniProt sequence of the peptide after adding either 50 or 100 residues of context on either end of the peptide. For the prediction, the MSA was sliced to the region corresponding to the PDB-delimited peptide. MSAs were generated using colabfold, which made API calls to the mmseqs2 server requesting a paired MSA only. Predictions were made on the paired MSAs of depth at least 50 and average Jensen-Shannon divergence of the peptide region of at least 0.3.

Calculating conservation and hydrophobicity
Conservation was calculated using Jensen-Shannon divergence, which compares the residue frequencies in a column of an MSA to a background distribution of residue frequencies (here, BLOSUM62) [27]. The hydrophobicity of binding site residues was calculated based on the Kyte-Doolittle hydrophobicity scale as provided in Biopython [28].

Visualization
Upset plots were created with https://github.com/jnothman/UpSetPlot [29]. Protein structure images were created with PyMol v2.5.0 Open-Source.

Supplementary References

1. JANIN, J. 2005. Assessing predictions of protein-protein interaction: the CAPRI experiment. *Protein Science* 14:278–283.
2. MIRABELLO, C. AND WALLNER, B. 2024. DockQ v2: improved automatic quality measure for protein multimers, nucleic acids, and small molecules. *Bioinformatics* 40:btae586.
3. BASU, S. AND WALLNER, B. 2016. DockQ: A Quality Measure for Protein-Protein Docking Models. *PLOS One* 11:e0161879.
4. BRYANT, P., POZZATI, G., AND ELOFSSON, A. 2022. Improved prediction of protein-protein interactions using AlphaFold2. *Nature Communications* 13:1265.
5. PDB 2016. Crystal structure of a bacterial signalling complex.
6. LI, S., LI, T., XU, Y., ZHANG, Q., ZHANG, W., CHE, S., LIU, R., WANG, Y., AND BARTLAM, M. 2015. Structural insights into YfiR sequestering by YfiB in Pseudomonas aeruginosa PAO1. *Scientific Reports* 5:16915–16915.
7. TSABAN, T., VARGA, J. K., AVRAHAM, O., BEN-AHARON, Z., KHRAMUSHIN, A., AND SCHUELER-FURMAN, O. 2022. Harnessing protein folding neural networks for peptide–protein docking. *Nature Communications* 13:176.
8. KO, J. AND LEE, J. 2021. Can AlphaFold2 predict protein-peptide complex structures accurately? *bioRxiv* p. 2021.07.27.453972.
9. BRET, H., GAO, J., ZEA, D. J., ANDREANI, J., AND GUEROIS, R. 2024. From interaction networks to interfaces, scanning intrinsically disordered regions using AlphaFold2. *Nature Communications* 15:597.
10. JOHANSSON-ÅKHE, I. AND WALLNER, B. 2022. Improving peptide-protein docking with AlphaFold-Multimer using forced sampling. *Frontiers in Bioinformatics* 2.
11. LEE, C. Y., HUBRICH, D., VARGA, J. K., SCHÄFER, C., WELZEL, M., SCHUMBERA, E., DJOKIC, M., STROM, J. M., SCHÖNFELD, J., GEIST, J. L., POLAT, F., GIBSON, T. J., KELLER VALSECCHI, C. I., KUMAR, M., SCHUELER-FURMAN, O., AND LUCK, K. 2024. Systematic discovery of protein interaction interfaces using AlphaFold and experimental validation. *Molecular Systems Biology* 20:75–97.
12. ZHOU, F., GUO, S., PENG, X., ZHANG, S., MEN, C., DUAN, X., ZHU, G., WANG, Z., LI, W., MU, Y., ZHENG, L., LIU, H., AND WANG, S. 2025. Benchmarking AlphaFold3-like Methods for Protein-Peptide Complex Prediction. *bioRxiv* p. 2025.03.09.642277.
13. COCK, P. J. A., ANTAO, T., CHANG, J. T., CHAPMAN, B. A., COX, C. J., DALKE, A., FRIEDBERG, I., HAMELRYCK, T., KAUFF, F., WILCZYNSKI, B., AND DE HOON, M. J. L. 2009. Biopython: freely available Python tools for computational molecular biology and bioinformatics. *Bioinformatics* 25:1422–1423.
14. SCHRÖDINGER, LLC. The PyMOL Molecular Graphics System, Version 3.1.3.
15. MARTINS, P. M., SANTOS, L. H., MARIANO, D., QUEIROZ, F. C., BASTOS, L. L., GOMES, I. D. S., FISCHER, P. H. C., ROCHA, R. E. O., SILVEIRA, S. A., DE LIMA, L. H. F., DE MAGALHÃES, M. T. Q., OLIVEIRA, M. G. A., AND DE MELO-MINARDI, R. C. 2021. Propedia: a database for protein–peptide identification based on a hybrid clustering algorithm. *BMC Bioinformatics* 22:1.
16. MARTINS, P., MARIANO, D., CARVALHO, F. C., BASTOS, L. L., MORAES, L., PAIXÃO, V., AND CARDOSO DE MELO-MINARDI, R. 2023. Propedia v2.3: A novel representation approach for the peptide-protein interaction database using graph-based structural signatures. *Frontiers in Bioinformatics* 3.
17. LEE, B. AND RICHARDS, F. M. 1971. The interpretation of protein structures: estimation of static accessibility. *Journal of Molecular Biology* 55:379–400.
18. VAN KEMPEN, M., KIM, S. S., TUMESCHEIT, C., MIRDITA, M., LEE, J., GILCHRIST, C. L. M., SÖDING, J., AND STEINEGGER, M. 2024. Fast and accurate protein structure search with Foldseek. *Nature Biotechnology* 42:243–246.
19. MIRDITA, M., SCHÜTZE, K., MORIWAKI, Y., HEO, L., OVCHINNIKOV, S., AND STEINEGGER, M. 2022. ColabFold: making protein folding accessible to all. *Nature Methods* 19:679–682.
20. MORIWAKI, Y. 2024. LocalColabFold. Publication Title: GitHub repository.
21. BENNETT, N. R., COVENTRY, B., GORESHNIK, I., HUANG, B., ALLEN, A., VAFEADOS, D., PENG, Y. P., DAUPARAS, J., BAEK, M., STEWART, L., DIMAIO, F., DE MUNCK, S., SAVVIDES, S. N., AND BAKER, D. 2023. Improving de novo protein binder design with deep learning. *Nature Communications* 14:2625.
22. KABSCH, W. AND SANDER, C. 1983. Dictionary of protein secondary structure: Pattern recognition of hydrogen-bonded and geometrical features. *Biopolymers* 22:2577–2637.
23. JOOSTEN, R. P., TE BEEK, T. A., KRIEGER, E., HEKKELMAN, M. L., HOOFT, R. W., SCHNEIDER, R., SANDER, C., AND VRIEND, G. 2011. A series of PDB related databases for everyday needs. *Nucleic Acids Research* 39:D411–D419.
24. AHDRITZ, G., BOUATTA, N., FLORISTEAN, C., KADYAN, S., XIA, Q., GERECKE, W., O'DONNELL, T. J., BERENBERG, D., FISK, I., ZANICHELLI, N., ZHANG, B., NOWACZYNSKI, A., WANG, B., STEPNIEWSKA-DZIUBINSKA, M. M., ZHANG, S., OJEWOLE, A., GUNEY, M. E., BIDERMAN, S., WATKINS, A. M., RA, S., LORENZO, P. R., NIVON, L., WEITZNER, B., BAN, Y.-E. A., CHEN, S., ZHANG, M., LI, C., SONG, S. L., HE, Y., SORGER, P. K., MOSTAQUE, E., ZHANG, Z., BONNEAU, R., AND ALQURAISHI, M. 2024. OpenFold: retraining AlphaFold2 yields new insights into its learning mechanisms and capacity for generalization. *Nature Methods* 21:1514–1524.
25. ZHANG, Y. AND SKOLNICK, J. 2005. TM-align: a protein structure alignment algorithm based on the TM-score. *Nucleic Acids Research* 33:2302–2309.
26. DANA, J. M., GUTMANAS, A., TYAGI, N., QI, G., O'DONOVAN, C., MARTIN, M., AND VELANKAR, S. 2019. SIFTS: updated Structure Integration with Function, Taxonomy and Sequences resource allows 40-fold increase in coverage of structure-based annotations for proteins. *Nucleic Acids Research* 47:D482–D489.
27. CAPRA, J. A. AND SINGH, M. 2007. Predicting functionally important residues from sequence conservation. *Bioinformatics* 23:1875–1882.
28. KYTE, J. AND DOOLITTLE, R. F. 1982. A simple method for displaying the hydropathic character of a protein. *Journal of Molecular Biology* 157:105–132.
29. A. LEX, N. GEHLENBORG, H. STROBELT, R. VUILLEMOT, AND H. PFISTER 2014. UpSet: Visualization of Intersecting Sets. *IEEE Transactions on Visualization and Computer Graphics* 20:1983–1992.
